# Chemical Distance Measurement and System Pharmacology Approach Uncover the Novel Protective Effects of Biotransformed Ginsenoside C-Mc against UVB-Irradiated Photoaging

**DOI:** 10.1155/2022/4691576

**Published:** 2022-02-09

**Authors:** Xiao-yi Liu, Hui Li, Eunson Hwang, Bom Park, Yong-kun Xiao, Senmiao Liu, Jiansong Fang, Yeon-Ju Kim, Tae-Hoo Yi, Chuipu Cai

**Affiliations:** ^1^School of Basic Medical Sciences, Guangzhou University of Chinese Medicine, Guangzhou, China; ^2^Division of Data Intelligence, Department of Computer Science, Key Laboratory of Intelligent Manufacturing Technology of Ministry of Education, College of Engineering, Shantou University, Shantou, China; ^3^Department of Oriental Medicinal Biotechnology, College of Life Sciences, Kyung Hee University, Republic of Korea; ^4^Science and Technology Innovation Center, Guangzhou University of Chinese Medicine, Guangzhou, China

## Abstract

Long-term exposure to ultraviolet light induces photoaging and may eventually increase the risk of skin carcinogenesis. Rare minor ginsenosides isolating from traditional medicine Panax (ginseng) have shown biomedical efficacy as antioxidation and antiphotodamage agents. However, due to the difficulty of component extraction and wide variety of ginsenoside, the identification of active antiphotoaging ginsenoside remains a huge challenge. In this study, we proposed a novel *in silico* approach to identify potential compound against photoaging from 82 ginsenosides. Specifically, we calculated the shortest distance between unknown and known antiphotoaging ginsenoside set in the chemical space and applied chemical structure similarity assessment, drug-likeness screening, and ADMET evaluation for the candidates. We highlighted three rare minor ginsenosides (C-Mc, Mx, and F2) that possess high potential as antiphotoaging agents. Among them, C-Mc deriving from American ginseng (*Panax quinquefolius* L.) was validated by wet-lab experimental assays and showed significant antioxidant and cytoprotective activity against UVB-induced photodamage in human dermal fibroblasts. Furthermore, system pharmacology analysis was conducted to explore the therapeutic targets and molecular mechanisms through integrating global drug-target network, high quality photoaging-related gene profile from multiomics data, and skin tissue-specific expression protein network. In combination with *in vitro* assays, we found that C-Mc suppressed MMP production through regulating the MAPK/AP-1/NF-*κ*B pathway and expedited collagen synthesis via the TGF-*β*/Smad pathway, as well as enhanced the expression of Nrf2/ARE to hold a balance of endogenous oxidation. Overall, this study offers an effective drug discovery framework combining *in silico* prediction and *in vitro* validation, uncovering that ginsenoside C-Mc has potential antiphotoaging properties and might be a novel natural agent for use in oral drug, skincare products, or functional food.

## 1. Introduction

Cutaneous aging is a multisystem degenerative process characterized by diverse alterations in physiological properties that caused by intrinsic and extrinsic factors. Intrinsic aging occurs as a consequence of hereditary physiological adaptations, and extrinsic aging is mediated by exterior influences such as environmental factors, ultraviolet (UV) exposure, excessive alcohol, and immoderate repetitive muscle use [1, 2]. UV irradiation is the dominant factor in extrinsic aging and can result in hyperpigmentation, wrinkled skin, increased roughness, and loss of elasticity [3]. Previous studies indicated that chronic exposure to UVB radiation could induce skin damage by increasing the levels of reactive oxygen species (ROS) and eventually cause characteristic symptoms of skin photoaging, such as hyperplasia of sebaceous glands, deeper and wider wrinkle formation, and reduction in skin elasticity [4–7]. Moreover, there is clear evidence that the development of benign and malignant neoplasms is increased on photoaged skin [8]. Thus, it is a need to develop novel and effective agents against photoaging.

Medicinal Panax (ginseng) herbs, such as Asian ginseng (*Panax ginseng* C.A. Meyer) and American ginseng (*Panax quinquefolius* L.), are always the top-selling natural health products that have been promoted as a panacea or “cure-all” [9]. As traditional herbal medicine, Panax has a long-term clinical application in dermatological diseases and been mechanistically investigated for its therapeutic effects [10]. Specifically, ginsenosides are the principal constituents responsible for the biological activities of Panax and have been approved for use in various active pharmaceuticals due to their immunoregulatory, anti-inflammatory, antitumor, antiaging, and skin whitening effects [11–14]. Ginsenosides are triterpenoid saponin groups that are classified as dammarane type and oleanane type according to the aglycone skeleton. The dammarane type ginsenosides can be further divided into two groups: PPD-type ginsenosides and protopanaxatriol type ginsenosides [15]. Additionally, previous studies have shown that pharmacological activity is related to the modified glycosylated chain and increases with a reduction in the number of sugar moieties [16]. Recently, the rare minor ginsenosides have been proved to be more efficient active pharmacological agents compared with the major ginsenosides [17]. Accordingly, many rare ginsenosides such as Rg3 and C-Mx have been demonstrated pharmacological activities with regard to UVB-induced skin aging [18, 19]. Until now, hundreds of rare ginsenosides have been identified. However, most of them are extremely difficult to extract or efficient produce. Therefore, considering the labor and time cost, it is inevitable a huge challenge to comprehensively evaluate the protective effect against photoaging of these ginsenosides via conventional experimental assays.

In recent years, *in silico* approaches have been broadly applied in drug discovery and successfully identified novel drug candidates for the prevention and treatment of many diseases [20–22]. Ligand-based methods, such as quantitative structure-activity relationship (QSAR) models constructed by machine learning or deep learning algorithm, are the most common used approaches [23–25]. However, these approaches require high quality of training set containing sufficient and diverse samples, which is not suitable for the drug discovery of photoaging owing to the lack of enough experimental and medicinal data. Structure-based approaches such as molecular docking are always encumbered by the computing speed and the resolution of crystal structures [23]. Besides, network approach based on system pharmacology, which comprehensively considers the information of drug-target network and disease-related genes, provides new insights into the identification of active ingredients against disease [26–28]. Nevertheless, as the object of this study is rare ginsenosides, most of which remain nearly unknown for the information of interacted targets. Since current approaches could not be adequate for the requirements, it is necessary to design a novel efficient *in silico* strategy to identify the potential active agents for photodamage from various ginsenosides.

**Figure 1:**
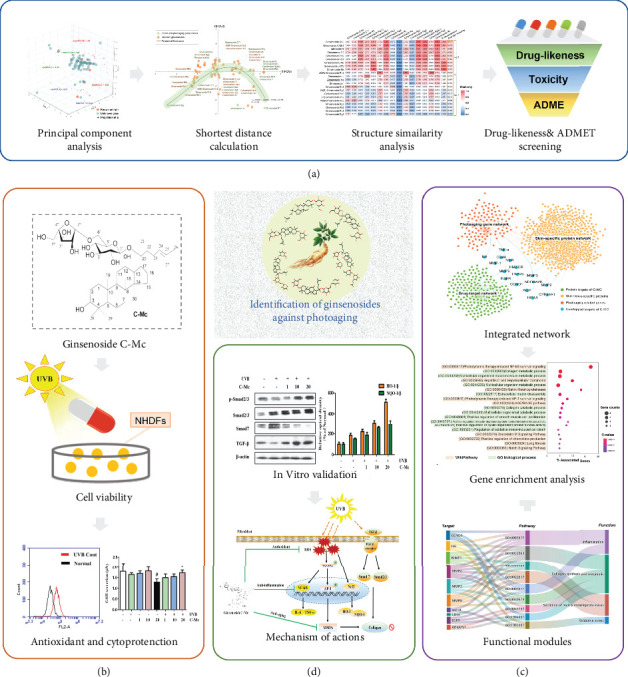
Schematic diagram illustrating *in silico* methodology and *in vitro* validation for identification of active ginsenosides against photoaging. (a) *In silico* identification of antiphotoaging candidates from 82 ginsenosides. (b) *In vitro* validation of antiphotoaging effects for the promising candidate ginsenoside C-Mc. (c) System pharmacology-based exploration of the potential therapeutic targets, biological process, signal pathway, and regulatory function. (d) *In vitro* assay for elucidating the mechanism actions of ginsenoside C-Mc against photoaging.

In this study, we proposed an *in silico* and *in vitro* integrated framework for identification of active ginsenosides against photoaging (Figure 1 and Supplementary Figure S1). Specifically, we first designed a novel drug virtual screening protocol, which consists of shortest distance measurement in the chemical space of principle component analysis, pairwise chemical structure similarity analysis, drug-likeness screening, and ADMET (adsorption, distribution, metabolism, excretion, and toxicity) evaluation (Figure 1(a)). Based on the computational prediction, the rare minor ginsenoside C-Mc was selected to evaluate its protective effect towards UVB-irradiated photodamage in human skin dermal fibroblasts (Figure 1(b)). After that, system pharmacology-based analysis, including construction of integrated network, gene enrichment analysis, and functional module induction, was performed to explore the potential therapeutic targets, biological process, signal pathway, and regulatory function (Figure 1(c)). Finally, further *in vitro* experiments were conduct to validate the predictions and systematically elucidate the antiphotoaging molecular mechanism of C-Mc (Figure 1(d)).

## 2. Materials and Methods

### 2.1. In Silico Experiments

#### 2.1.1. Collection of Ginsenosides

The information and structures of ginsenosides were collected from literatures and PubChem database of National Center for Biotechnology Information (NCBI). These compounds were further converted to unified InChiKey and Smiles format using Openbabel tool [29]. Totally, 98 ginsenosides with unique structure were obtained. Through consulting literature materials, 16 of them with previous reported antiphotoaging or photoprotective effects are labeled as known antiphotoaging agents, while the remaining 82 compounds were selected as unknown objects for screening (Supplementary Table S1).

#### 2.1.2. Molecular Processing and Principal Component Analysis (PCA)

The collected ginsenosides were processed by molecular washing and energy minimizing for protonating strong bases, deprotonating strong acids, removing inorganic counter ions, adding hydrogen atoms, and generating stereo isomers and valid single 3D conformers using MOE 2010 [30]. Furthermore, two-dimensional (2D) descriptors were generated by MOE 2010 to represent molecular properties and structural information. The MOE descriptor set consists of 186 features, covering physical property, pharmacophore feature, atom count and bond count, adjacency and distance matrix, subdivided surface area, Kier and Hall connectivity and Kappa shape indices, and partial charge descriptors. After that, principal component analysis (PCA) was performed by MOE to reduce the dimensionality of molecular descriptors by linearly transforming the data. The 186 molecular descriptors were used as input variables. The detailed information of the PCA computational procedure can be found in the following references [30, 31].

#### 2.1.3. Shortest Distance Measurement among Compounds in the PCA Space

The first two outputs of PCA for each ginsenoside were defined as its position in the two-dimensional PCA space. Each point in the coordinate represents a ginsenoside. We hypothesize that a ginsenoside without previous antiphotoaging reports exhibits a high possibility to possess photoprotective effects if its position in the PCA space is relatively close to those of the known antiphotoaging ginsenosides. Here, the points of the 16 known antiphotoaging ginsenosides were first fitted as a curve by a polynomial. Then, the shortest distance (*d*) between each point of unknown ginsenoside *Q*(*x*_0_, *y*_0_) and each point on the fitted curve of the known ginsenosides *f*(*x*, *y*) = 0 was calculated, as the Euclidean distance metric described below:

(1) $$\begin{array}{c} d=\sqrt{{\left(x-x_{0}\right)}^{2}+{\left(y-y_{0}\right)}^{2}}. \end{array}$$

Demand that

(2) $$\begin{array}{c} L\left(x,y\right)={\left(x-x_{0}\right)}^{2}+{\left(y-y_{0}\right)}^{2}+\gamma f\left(x,y\right). \end{array}$$

The following equations could be obtained by Lagrange multipliers:

(3) $$\begin{array}{c} \left\{\begin{array}{c} L_{x}\left(x,y\right)=2\left(x-x_{0}\right)+\gamma f_{x}\left(x,y\right)=0,\\ L_{y}\left(x,y\right)=2\left(y-y_{0}\right)+\gamma f_{y}\left(x,y\right)=0,\\ f\left(x,y\right)=0, \end{array}\right. \end{array}$$

where *fx*(*x*, *y*) and *fy*(*x*, *y*) are the partial derivatives of *f*(*x*, *y*) with respect to *x* and *y*. By applying equation set (3), the corresponding point *P*(*x*, *y*) on the curve is obtained, and the shortest distance *d*_min_ between *P* and *Q* can be calculated by the Euclidean distance Equation (1). Assume the endpoints of curve are *M* and *N*, the shortest distance *D*_min_ between point *Q* and the fitted curve is the minimum value among *d*_min_(*Q*, *P*), *d*_min_(*Q*, *M*), and *d*_min_(*Q*, *N*), which can be described as follows:

(4) $$\begin{array}{c} D_{\min}=\min\left\{\begin{array}{c} d_{\min}\left(Q,P\right),\\ d_{\min}\left(Q,M\right),\\ d_{\min}\left(Q,N\right). \end{array}\right. \end{array}$$

In this study, according to the domain of chemical space, the shortest distance smaller than 0.2 is considered as significant.

#### 2.1.4. Pairwise Chemical Structure Similarity Analysis

In addition to the PCA based on chemical molecular properties, the chemical similarity analysis based on chemical structures was also applied to measure the distance between each pair of known and unknown antiphotoaging ginsenosides. The fp_topological_4 fingerprint was selected to represent the structures of ginsenosides, while Tanimoto metric was used to calculate the similarity index between each other, as described below:

(5) $$\begin{array}{c} \text{Tanimoto similarity}=\frac{c}{\left(a+b-c\right)}, \end{array}$$

where *a* and *b* represent the bits (number of fingerprint descriptors) in chemical structure of Cmp(*m*) and Cmp(*n*) and *c* refers to the bits coexist in both Cmp(*m*) and Cmp(*n*). A higher Tanimoto index means a higher similarity of their structures.

#### 2.1.5. Drug-Likeness Evaluation and ADMET Screening

In this study, the drug-likeness evaluation and ADMET screening were utilized to further exclude the candidates with undesirable pharmacokinetic properties and unacceptable toxicity. Specifically, the drug-likeness predictive model is provided by Dong's study, which was constructed by the machine learning algorithms of random forest (RF) with molecular descriptors of MACCS on a training set containing 6,731 positive and 6,769 negative samples [32]. Besides, pan assay interference compounds (PAINS), one of the most famous frequent hitters filters, was applied to screen potential false positive hits [33]. The ADMET properties, including human oral bioavailability 20% (*F*_20%_), plasma protein binding (PPB), rat acute oral toxicity (AOT), and skin sensitization, were evaluated by ADMETlab 2.0, a platform that integrated a series of well-performed predictive models for pharmacokinetics and toxicity [34].

#### 2.1.6. Construction of Drug-Target (D-T) Network of Ginsenoside C-Mc

Since currently there are no reported protein targets regulated by ginsenoside C-Mc, the D-T network was constructed through integrating predicted drug-target interactions (DTIs) obtained by the following ways: (1) target identification based on molecular docking simulation. The crystal structures of interested protein targets were retrieved from Protein DataBank (PDB), optimized in ChemBioOffice 2010 tool package, and implemented molecular docking with C-Mc according to the available standard Autodock protocol [35, 36]. We found C-Mc showed higher binding affinities to receptors MAPK, NF-*κ*B, IL-6, and TNF-*α* compared with control ligands (Supplementary Figure S2 and Table S2); (2) manually collection of reported targets of other ginsenosides from relevant literatures and our previous integrated DTI database of natural product [37]. It is plausible to hypothesize that these targets are more likely to interact with C-Mc since the ginsenosides are similar in structure. A total of 128 targets were collected; (3) potential target proteins predicted by balanced substructure–drug–target network-based inference (bSDTNBI) methods, a computational approach that can predict potential targets for new chemical entities [38, 39]. The molecular fingerprint of Klekota-Roth and default parameters were used, and the top 100 predicted targets for each compound were preserved. Finally, the D-T network contains 222 DTIs after removing the duplicated interactions and non-*Homo* sapiens proteins (Supplementary Table S3).

#### 2.1.7. Photoaging-Related Gene Profile Integrating from Multiomics Data

We manually curated and integrated a skin photoaging-related gene set from multiple resources, including (1) PolySearch 2.0, a significantly improved text-mining system for discovering associations biomedical entities [38]. The disease name of “photoaging” was inputted as query key word; (2) RNA sequencing data of photodamaged skins from Genotype-Tissue Expression (GTEx) project. The transcriptomic changes induced by UV irradiation were characterized through differentially expressed gene analysis and weighted gene coexpression network analysis [40]; (3) differentially expressed genes screened by microarray analysis between sun-exposed (anterior ear skin) and sun-protected (retroauricular skin) skins of 6 patients with facial photoaging [41]; and (4) published skin photoaging-related study literatures from PubMed database (as of May 2021). All the collected proteins/genes were converted into unified *Homo* sapiens gene symbol name and Entrez ID based on the NCBI Gene Database (https://www.ncbi.nlm.nih.gov/gene). Finally, 124 unique photoaging-related genes were collected after removing the duplicated ones (Supplementary Table S4).

#### 2.1.8. Skin Tissue-Specific Expression Protein Network

The RNA-seq data (RPKM value) of skin tissue was extracted from the GTEx V6 release (https://gtexportal.org/home/). In this work, genes (*i*) with RPKM value ≥ 1 in over 80% of skin samples were considered as specific expressed in the skin tissue (*t*). The average expression *E*(*i*) and the standard deviation *δ*_*E*(*i*)_ of a gene's expression towards the skin tissue were calculated, and significance of gene (*i*) expression in the skin tissue (*t*) was quantified by the equation given below:

(6) $$\begin{array}{c} Z_{E\left(i,t\right)}=\frac{E\left(i,t\right)-E\left(i\right)}{\delta_{E\left(i\right)}}. \end{array}$$

We asserted that genes with *Z*-expression score larger than 2 were regarded as high expressed in the skin tissue. The information of the skin tissue-specific expression protein network containing 907 proteins is provided in Supplementary Table S5.

### 2.2. In Vitro Experiments

#### 2.2.1. Chemicals and Biochemical

Ginsenosides Rc and C-Mc were obtained from Ambo Laboratory (Dacejeon, Korea). ELISA kits for MMPs (MMP-1 and MMP-3), IL-6, TGF-*β*1, procollagen type I, and VEGF were purchased from R&D Systems, Inc. (Minneapolis, MN, USA). DMEM, FBS, and penicillin/streptomycin were purchased from Gibco BRL (Aidenbac, Germany). Glutathione content was assayed using a GSH assay kit (Cayman Chemical Co, Ann Arbor, MI, USA), and the lactate dehydrogenase (LDH) cytotoxicity assay kit was purchased from Roche Diagnostics GmbH (Roche Diagnostics, Mannheim, Germany). Antibodies were purchased from Santa Cruz Biotechnology (Santa Cruz, CA, USA) and Cell Science (Canton, MA, USA). Solvents were purchased from Samchun Pure Chemicals (Korea) unless otherwise stated.

#### 2.2.2. Preparation and Identification of Ginsenoside C-Mc

The minor ginsenoside C-Mc was prepared from American ginseng PPD ginsenosides, using 6% PPD ginsenoside substrate in acetate buffer (0.02 M and pH 5.0). Ginsenoside C-Mc was reacted with a volume of crude enzyme (*A. niger g.848* strain) in the bioreactor.

In brief, chromatographic analysis was completed by a Waters ACQUITY UPLC system using XBridge C-18 chromatographic column (5 *μ*m, *φ*4.6 × 250 mm) was used to analyze the samples. The mobile phase was acetonitrile (A) and water (B), column oven was 35°C, and flow rate is 0.6 ml/min. Detection wavelength of the DAD was 203 nm.

The mass examination was completed by a Waters SQ detector through a positive electrospray ionization pattern. Briefly, cone gas flow was set at 50 L/h, and desolvation gas flow was 550 L/h. Capillary voltage and cone voltage were 3.5 kV and 30 V, respectively.

The sample of ginsenoside C-Mc was identified as 20-O-[*α*-L-arabinofuranosyl-(1→6)-*β*-D-glucopyranosyl]-20(S)-protopanaxdiol using a BrukeAvance 600 (^1^H: 600 MHz and ^13^C: 150 MHz) NMR spectrometer (Switzerland) in our previous study [42].

#### 2.2.3. Cell Culture, UVB Irradiation, and Ginsenoside C-Mc Treatment

Normal human dermal fibroblasts (NHDFs) were obtained from a skin biopsy healthy male donor (MCTT, Seoul, Korea) and grown in DMEM (1% penicillin-streptomycin and 10% heat-inactivated FBS) in 5% CO_2_ incubator at 37°C. In brief, experimental groups were exposed to UVB irradiation at 144 mJ/cm^2^ using a Bio-Link BLX-312 machine (Vilber Lourmat GmbH, France) and then treated with 1, 10, or 20 *μ*M ginsenoside C-Mc. Control groups were subjected to the same protocol without exposure to UVB radiation.

#### 2.2.4. Cell Viability Assay

The effect of ginsenoside C-Mc on viability of NHDFs was evaluated by MTT assay. After treatment for 72 h, 1 mL of medium was removed for ELISA. MTT solution (100 *μ*g/ml MTT in PBS) was added to the remaining medium followed by incubation at 37°C for 4 h. After removal of the medium, 800 *μ*L DMSO was added to each well and the absorbance was detected at 595 nm.

#### 2.2.5. Measurement of ROS Scavenging Ability

After UVB irradiation (144 mJ/cm^2^), NHDFs were washed twice with PBS and treated with ginsenoside C-Mc (1, 10, and 20 *μ*M) for 24 h. The cells were stained with 30 mM 2⁣′7⁣′-dichlorofluorescein diacetate (DCFH-DA; Sigma-Aldrich) for 30 min at 37°C and then analyzed by flow cytometry (BD Accuri C6; Becton-Dickinson, San Jose, CA, USA).

#### 2.2.6. Intracellular GSH Determination

The level of GSH reductase was tested using a GSH assay kit (Cayman Chemical Co, Ann Arbor, MI). Briefly, the sulfhydryl group of GSH reacts with 5,5⁣′-dithiobis-2-nitrobenzoic acid (DTNB) to yield the product 5-thio-2-nitrobenzoic acid (TNB). The final absorbance was detected at 405 nm.

#### 2.2.7. Production of MMP-1, IL-6, MMP-3, Procollagen Type I, TGF-*β*1, and VEGF

The concentrations of MMP-1, IL-6, MMP-3, procollagen type I, TGF-*β*1, and VEGF in cell medium were quantified using commercially available ELISA kits as described above. Each experiment was repeated at least three times.

#### 2.2.8. Lactate Dehydrogenase (LDH) Release

A LDH cytotoxicity assay kit (Roche Diagnostics, Mannheim, Germany) was used to measure the LDH level. In this assay, LDH reduces NAD to NADH, which then interacts with a specific probe to produce a colored product. After incubation for 40 min at 37°C, the absorbance was detected at 450 nm.

#### 2.2.9. Quantitative Real-Time RT-PCR

NHDFs were harvested after UVB irradiation (144 mJ/cm^2^) and treated with ginsenoside C-Mc (1, 10, and 20 *μ*M). RNA was isolated from cells using TRIZOL reagent according to the manufacturer's instructions (Invitrogen Life Technologies, Carlsbad, CA). The expression of MMP-1, MMP-3, procollagen type I, iNOS, TNF-*α*, and IL-6 mRNA was measured by real-time PCR using SYBR^@^ Green master mix in a BioRad CFX Connect Real-Time PCR Detection System (BioRad, Hercules, CA). GAPDH was used for internal normalization. All experiments were carried out in triplicate. The primer sequences are provided in Supplementary Table S6.

#### 2.2.10. Cytosolic and Nuclear Extracts

Cells were harvested after UVB irradiation (144 mJ/cm^2^), and the protein was extracted. Cytosolic and nuclear fractions were separated using a commercial kit (NE-PER nuclear and cytoplasmic extraction reagents; Pierce).

#### 2.2.11. Western Blot Analysis

Western blotting was performed to detect components of the MAPK/AP-1, Nrf2/ARE, TGF-*β*/Smad, and NF-*κ*B/I*κ*B-*α* signaling pathways. In brief, cells were lysed with RIPA buffer (Cell Signaling Technology, USA) and centrifuged. Protein concentration was measured using Bradford reagent, and equal amounts of protein for each sample were separated by 8% or 10% SDS-PAGE and subjected to immunoblotting using a standard protocol and an ECL Western blot detection system (Amersham Pharmacia Biotech, NJ, USA).

### 2.3. Statistical Analysis

All data were based on three independent experiments. Data were expressed as mean ± SD using GraphPad Prism 5 (GraphPad Software, Inc., CA, USA). Statistical comparisons between different treatments were performed using a one-way analysis of variance (ANOVA) and Student's *t*-tests. Statistical significance was set at *p* < 0.05.

## 3. Results

### 3.1. Preliminary Identification of Potential Antiphotoaging Ginsenosides Based on Shortest Distance Calculation in PCA Space

**Figure 2:**
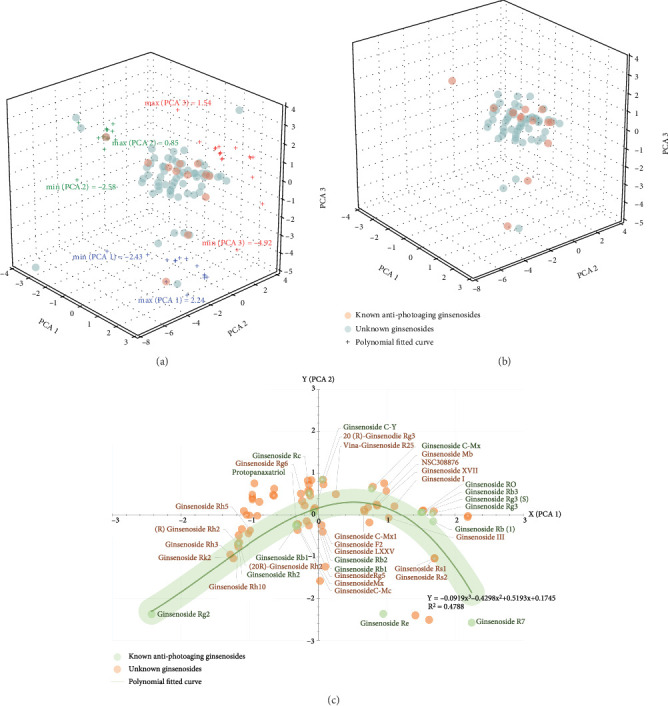
Preliminary identification of potential antiphotoaging ginsenosides through shortest distance measurement among compounds in the PCA space. (a) Calculation of the rational boundary constraint of the space of PCA. (b) Eliminating the improper compounds outside the boundary constraint of the space. (c) Shortest distance calculation between known and unknown antiphotoaging ginsenosides.

On the basis of the 16 known antiphotoaging ginsenosides, we firstly figure out the rational boundary constraint of the space of PCA (Figure 2(a)). The maximum and minimum values of PCA 1, PCA 2, and PCA 3 of the 16 ginsenosides, respectively, were set as the thresholds for eliminating the improper compounds. As shown in Figure 2(b), 15 of the 82 candidate ginsenosides were removed in this step. After that, the 16 known antiphotoaging ginsenosides were fitted as a curve by a polynomial of third order in the PCA coordinate frame, and the shortest distance between each point of unknown ginsenoside and the fitted curve was calculated (Figure 2(c)). We identified 22 ginsenosides have a significant shortest distance (*D*_min_ < 0.2) to the set of known active ginsenosides, which are preliminary identified as potential candidates for further study (Supplementary Table S7).

### 3.2. Candidates Prioritizing via Pairwise Chemical Structure Similarity Analysis

**Figure 3:**
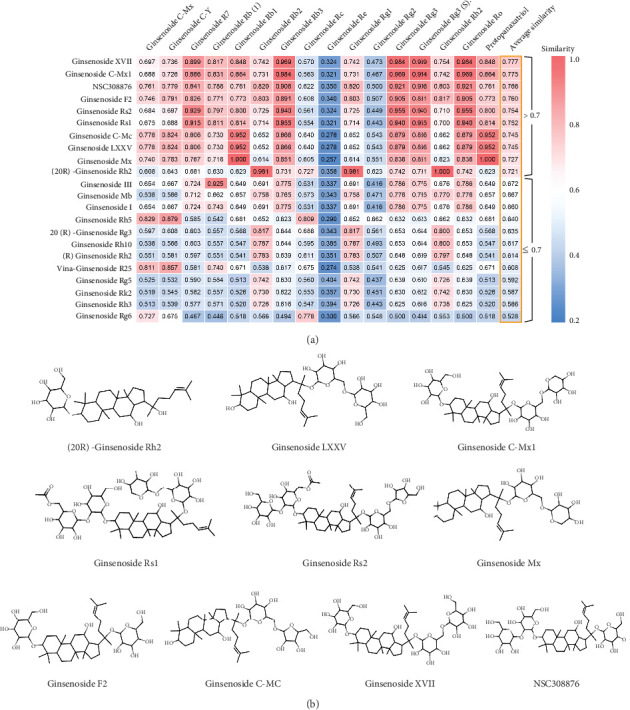
Pairwise chemical structure similarity analysis between known and unknown antiphotoaging ginsenosides. (a) Heatmap illustrating the similarity analysis results of the 16 known active ginsenosides and the 22 unknown ginsenosides. (b) Chemical structures of the top 10 ginsenosides with average similarity index higher than 0.7.

Compared to the above PCA based on molecular properties, the chemical similarity analysis conducting by molecular fingerprint can further screen out the more promising antiphotoaging ginsenosides from the perspective of compound structures. In this study, the similarity between the 16 known active ginsenosides and the remaining 22 unknown ginsenosides was measured individually. The average similarity index was calculated for each unknown ginsenosides by averaging the similarity results of 16 pairs of compounds. As presented in Figure 3(a), there are 10 ginsenosides possessing an average similarity index higher than 0.7, including ginsenosides C-Mc, XVII, C-Mx1, F2, Rs2, LXXV, Mx, Rs1, (20R)-ginsenoside Rh2, and NSC308876, which are supposed to have higher possibilities as photoprotective agents. The structures of the 10 ginsenosides are provided in Figure 3(b).

### 3.3. Drug-Likeness Evaluation and ADMET Screening for the Candidates

It is widely recognized that drug-likeness evaluation and ADMET screening are the essential steps in the early stage of drug development that help to avoid late-stage failures [43, 44]. Thus, before the wet-lab experiments, a comprehensive *in silico* evaluation was conducted on the 10 predicted antiphotoaging ginsenosides. As listed in Table 1, all of them were predicted to be positive by the drug-likeness model and had passed the PAINS frequent hitter filters. They also showed low toxicity according to the results of AOT and skin sensitization prediction. For PPB, one of the major indicators reflecting the ability of drug uptake and distribution, these candidates except (20R)-ginsenoside Rh2 were considered to have a proper property with predicted value < 90%. However, in the evaluation of human oral bioavailability (*F*_20%_), six of the ten ginsenosides were labeled as poor that may have low efficiency of the drug delivery to the systemic circulation. Put together, there are three ginsenosides (C-Mc, F2, and Mx) have passed all the evaluation of drug-likeness and ADMET properties, which could be served as the most promising antiphotoaging drug candidates of ginsenosides.

### 3.4. In Vitro Validation of the Cytoprotective Activity of Ginsenoside C-Mc against UVB-Irradiated Photodamage

**Figure 4:**
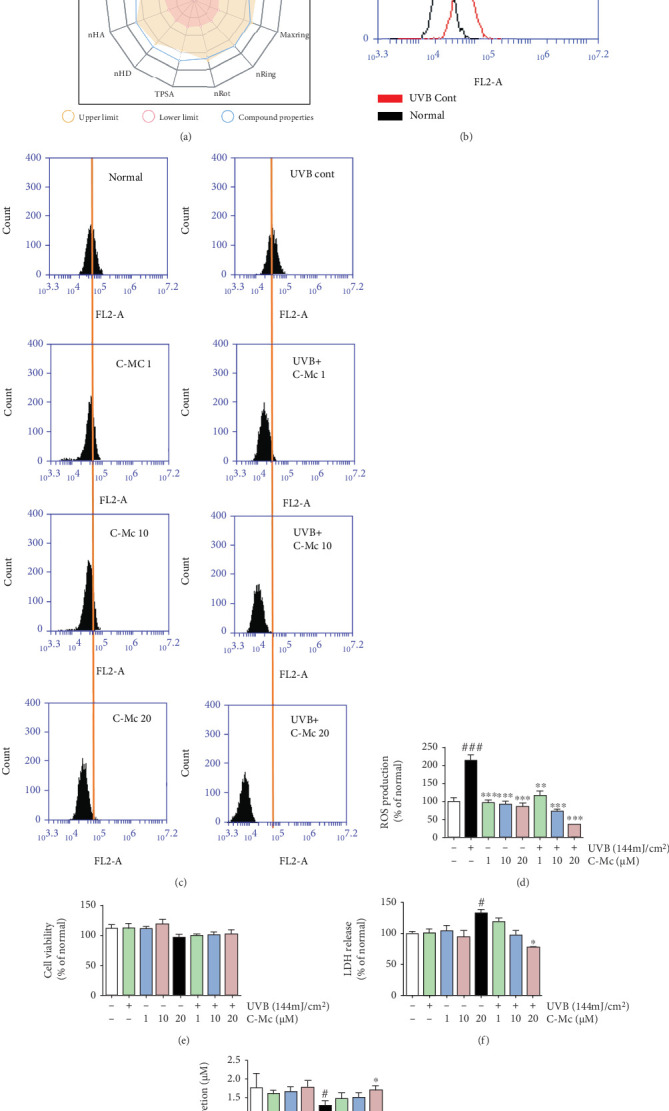
Free radical scavenging ability and cell viability of ginsenoside C-Mc. (a) Compound structure and physicochemical properties of ginsenoside C-Mc. The proper range of the chemical physicochemical properties is given by ADMETlab 2.0 [34]. (b–d) ROS production. (e) Cell viability. (f) LDH release. (g) GSH secretion. NHDFs were irradiated or nonirradiated with 144 mJ/cm^2^ UVB, followed by treatment with the indicated of ginsenoside C-Mc (1, 10, and 20 *μ*M). All data are shown as the mean ± SD of three independent experiments. # and ∗ indicate significant differences from the nonirradiated control and UVB-irradiated control groups. ^#^*p* < 0.05, ^##^*p* < 0.01, and ^###^*p* < 0.001 contrast with the nonirradiated control. ⁣^∗^*p* < 0.05, ⁣^∗∗^*p* < 0.01, and ⁣^∗∗∗^*p* < 0.001 contrast with the UVB-irradiated control. ⁣^∗^*p* < 0.05, ⁣^∗∗^*p* < 0.01, and ⁣^∗∗∗^*p* < 0.001.

As the *in silico* prediction suggested, ginsenosides C-Mc, F2, and Mx possess high potential as novel antiphotoaging agents. Comprehensively considering the extractability and compound properties, we finally selected ginsenoside C-Mc to validate its photoprotective activity in NHDFs (Figure 4(a)).

*Confirmation of ginsenoside C-Mc*. The purity of ginsenoside C-Mc was over 95.02% as detected by HPLC (Supplementary Figure S3a). C-Mc yielded a [M+Na]^+^ at *m*/*z* 777 or [M+H]^+^ at *m*/*z* 755 and MS^2^ ions at 605 [M+H-Araf-H_2_O]^+^, 443 [M+H-Araf-Glc-H_2_O]^+^, and 425 [M+H-Araf-Glc-2H_2_O]^+^ (Supplementary Figure S3b) [45, 46].

*Antioxidant ability of C-Mc*. In general, UVB exposure increases ROS generation in cells. This phenomenon was reversed after treatment with C-Mc; at a concentration of 20 *μ*M C-Mc, the ROS levels were reduced by 88.96% (Figures 4(b)–4(d)).

*Toxicity of ginsenoside C-Mc in NHDFs*. An MTT assay was performed to test NHDF viability. As presented in Figures 4(e), C-Mc showed no toxicity with concentrations of 1, 10, and 20 *μ*M in nonirradiated and irradiated NHDFs. Interestingly, C-Mc treatment at concentrations as high as 20 *μ*M unexpectedly increased cell viability in the nonirradiated groups. The broader concentration range (1, 10, 20, 30, 50, 100, and 200 *μ*M) for toxicity measurement is provided in Supplementary Figure S4.

*Cytoprotective activity of C-Mc in UVB-irradiated NHDFs*. As shown in Figure 4(f), UVB-exposed groups showed significantly enhanced LDH release, whereas treatment with ginsenoside C-Mc reduced the release of LDH. Because of its antioxidant properties, glutathione (GSH) is an essential factor in maintaining cellular redox homeostasis and preventing UV-induced oxidative stress damage [47]. As expected, after UVB irradiation, the intracellular GSH was notably exhausted, while C-Mc (1, 10, and 20 *μ*M) treatment reversed this trend. In particular, 20 *μ*M C-Mc enabled recovery of GSH level to almost the control value (Figure 4(g)).

### 3.5. System Pharmacology Analysis for Exploration of the Molecular Mechanisms of C-Mc against Photoaging

**Figure 5:**
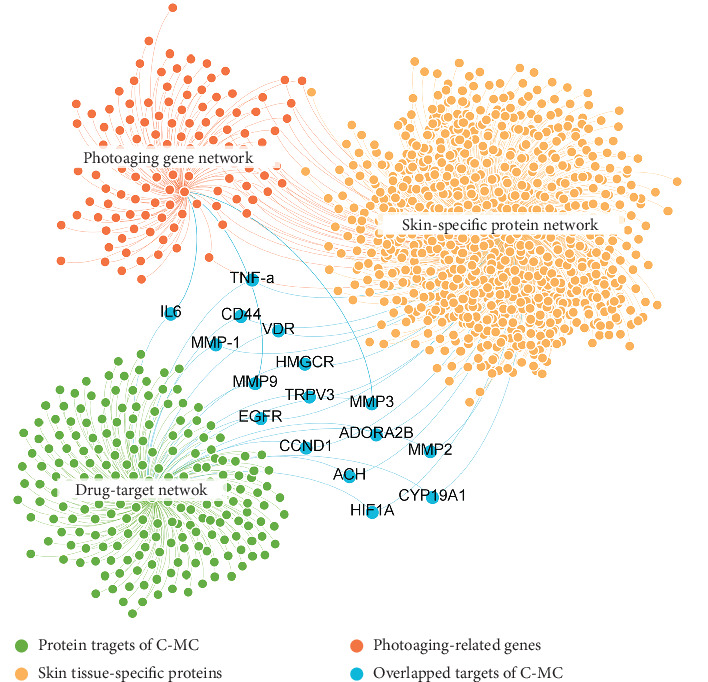
Global drug-target network of ginsenoside C-Mc interacted with photoaging-related gene network and skin tissue-specific expression protein network. Gene symbol names of the overlapped targets of C-Mc are displayed. The detailed information of the networks is provided in Supplementary Table S3-5.

**Figure 6:**
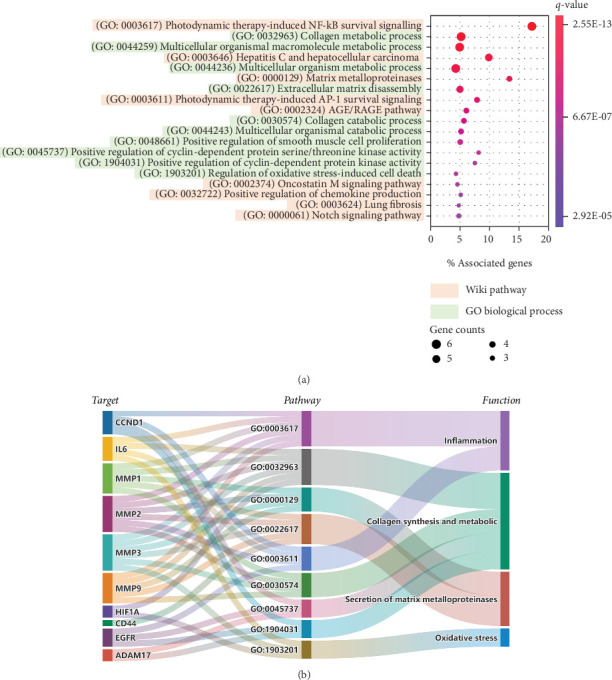
Gene enrichment analysis of the potential targets of ginsenoside C-Mc overlapped with photoaging and skin-specific genes. (a) Gene Ontology (GO) biological process enrichment and WikiPathway annotation; (b) Sankey diagram illustrating the relationship among the photoaging-relevant enriched pathways/processes, corresponding proteins, and biological function of C-Mc against photoaging.

There are currently no scientific data on the detailed molecular mechanism of the rare ginsenoside C-Mc in UVB-induced photoaging. Here, we performed system pharmacology analysis to explore the molecular mechanisms of C-Mc against photoaging through integrating drug-target network, photoaging-related gene network, and skin tissue-specific expression protein network. As shown in Figure 5, the D-T network contains 222 DTIs, of which 3 targets of C-Mc are interacted with photoaging-related protein network and 13 are connected to skin-specific protein network. The 16 targets overlapped with photoaging-related and skin-specific genes suggest the potential regulatory protein network of C-Mc against photoaging. We thus further conducted gene enrichment analysis on these overlapped targets using ClueGO plug-in of Cytoscape (v3.2.0) [48]. As presented in Figure 6(a), the gene enrichment analysis annotated 9 pathways from WikiPathways and 11 biological processes from Gene Ontology (GO) with corrected *p* values less than 0.01 (*q*, corrected with Bonferroni step down). We found that most of the enriched pathways/processes have high correlation with photodamage. For instance, it is reported that the secretion of matrix metalloproteinases (MMPs) associated with degradation of extracellular matrix (ECM) proteins promotes UVB radiation-irradiated photodamage [49], which is consistent with the predicted pathways such as GO:0032963 (collagen metabolic process, *q* = 4.35*E* − 10) and GO:0000129 (matrix metalloproteinases, *q* = 2.72*E* − 08). Besides, as annotated by WikiPathway, these potential targets of C-Mc are involved in photodynamic therapy-induced AP-1 (GO:0003611, *q* = 1.97*E* − 07) and NF-*κ*B (GO:0003617, *q* = 2.55*E* − 13) survival signaling pathway. Previous studies demonstrated that AP-1 and NF-*κ*B are essential for MMP-1 upregulation and ECM degradation [50, 51].

We further classified the photoaging-relevant enriched pathways/processes and corresponding proteins to elucidate the major biological function of C-Mc against photoaging (Figure 6(b)). Obviously, these pathways share a lot of common proteins such as MMPs (MMP-1, MMP-2, MMP-3, and MMP-9), indicating that C-Mc may exert multitarget synergistic antiphotoaging effect via acting on these potential key targets. The biological pathways and processes can be summarized into four important functional modules related to photoaging, including inflammation, collagen synthesis and metabolic, secretion of MMPs, and oxidative stress (Figure 6(b)). In fact, the four functional modules are intrinsically connected. UVB radiation induces secretion of MMPs and further leads to the loss of collagen in the dermal ECM via activation of the mitogen-activated protein kinases (MAPK)/activator protein-1 (AP-1) pathway [49]. AP-1 is responsible for activating the expression of MMPs, which cause degradation of ECM-related proteins, and synthesis of collagen and elastin [50]. Several studies have demonstrated that the TGF-*β*/Smad pathway regulates procollagen synthesis [52]. Furthermore, UV radiation stimulates NF-*κ*B proteins through suppression of the synthesis of I*κ*B-*α* [53]. Activated NF-*κ*B regulates the release of proinflammatory cytokines, which play a critical role in upregulation of MMP-1 and ECM degradation [51]. Moreover, in skin exposed to UVB, overexpression of proinflammatory cytokines such as TNF-*α*, iNOS, and IL-6 further activates NF-*κ*B by enhancing its translocation to the nucleus, resulting in overexpression of vascular endothelial growth factor (VEGF) [54]. In view of previous findings, anti-inflammation, antioxidative, expediting collagen synthesis and prevention metabolic, and inhibiting the secretion of MMPs might be considered as the underlying mechanism of actions of C-Mc to prevent photoaging damage.

### 3.6. Experimental Validation of the Antiphotoaging Mechanisms of Ginsenoside C-Mc in UVB-Irradiated NHDFs

As the system pharmacology analysis revealed ginsenoside C-Mc might exert antiphotoaging effect by influencing four major functional modules, including inflammation, collagen synthesis and metabolic, secretion of MMPs, and oxidative stress (Figure 6). In the following sections, we further conducted experiments in UVB-irradiated NHDFs under the guidance of system pharmacology predictions, to systematically elucidate the influence of C-Mc on these functional modules and relevant signal pathways, aiming to better understand the underlying mechanism of actions of C-Mc against photoaging.

### 3.7. C-Mc Prevented Secretion of MMP-1 and MMP-3 and Inhibited MAPK/AP-1 Signaling Pathway in UVB-Irradiated NHDFs

**Figure 7:**
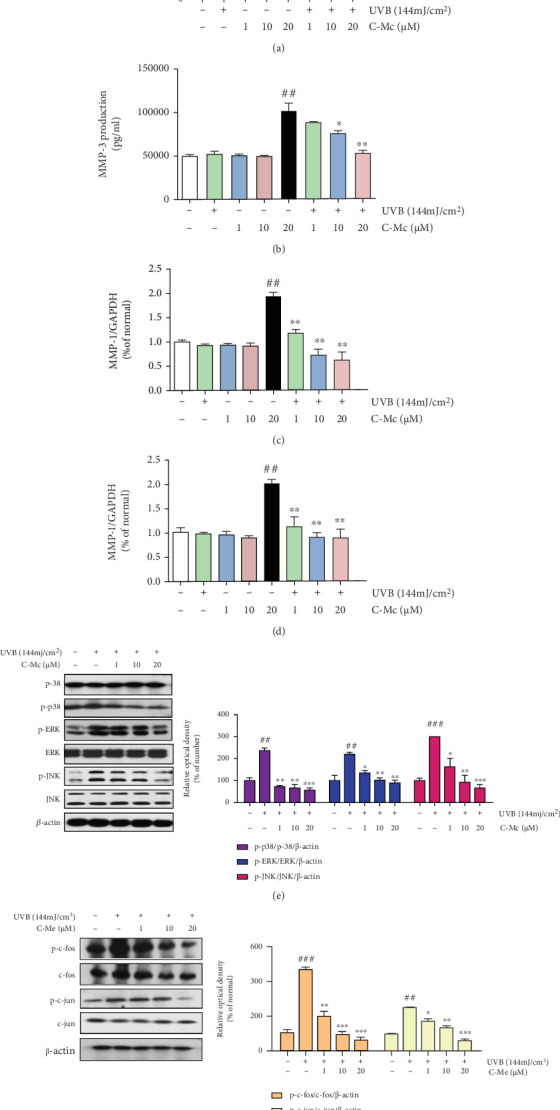
C-Mc prevented secretion of MMP-1 and MMP-3 and inhibited MAPK/AP-1 signaling pathway in UVB-irradiated NHDFs. Production of (a) MMP-1 and (b) MMP-3 under non-UVB irradiation and UVB-irradiated conditions; (c) MMP-1 and (d) MMP-3 mRNA expression. An equimolar quantity of mRNA was quantified compared to GAPDH. Cells were incubated in absence or presence of ginsenoside C-Mc at the present concentration after exposure to UVB radiation (144 mJ/cm^2^). (e) The protein levels of p38, ERK, and JNK in UVB-irradiated NHDFs measured by Western blot analysis. NHDFs were irradiated or nonirradiated with UVB, followed by treated with ginsenoside C-Mc for 1 h (MAPK). The signal intensities for phosphorylation levels of p38, ERK, and JNK. (f) The protein levels of c-Fos and c-Jun in UVB-irradiated NHDFs measured by Western blot analysis. NHDFs were irradiated or nonirradiated with UVB, followed by treatment with C-Mc for 4 h (AP-1). The signal intensities for phosphorylation levels of c-Fos and c-Jun. Values shown are the mean ± SD. # and ∗ indicate significant differences from the nonirradiated control and UVB-irradiated control groups. ^#^*p* < 0.05, ^##^*p* < 0.01, and ^###^*p* < 0.001 contrast with the nonirradiated control. ⁣^∗^*p* < 0.05, ⁣^∗∗^*p* < 0.01, and ⁣^∗∗∗^*p* < 0.001 contrast with the UVB-irradiated control.

System pharmacology analysis indicated that the secretion of MMPs is one of the major functional modules modulated by ginsenoside C-Mc. As shown in Figures 7(a) and 7(b), exposure of NHDFs to UVB irradiation (144 mJ/cm^2^) promoted secretion of MMP-1 and MMP-3, whereas treatment with C-Mc from 1 to 20 *μ*M significantly blocked MMP-1 and MMP-3 production. C-Mc decreased MMP-1 and MMP-3 secretion by 70.53% and 56.39%, respectively.

We evaluated MMP-1 and MMP-3 mRNA levels in UVB-exposed NHDFs by real-time PCR (Figures 7(c) and 7(d)). Our results indicated that the increased expression of MMP-1 and MMP-3 mRNAs induced by UVB irradiation was decreased by treatment with C-Mc. Specifically, 20 *μ*M C-Mc suppressed UVB-induced MMP-1 and MMP-3 expression by 68.35% and 49.55%, respectively. Moreover, 20 *μ*M C-Mc treatment upregulated procollagen type I expression by 198.36%. Consistent with the q-PCR data, ELISA results showed that expression of MMP-1 and MMP-3 proteins was strongly increased after UVB exposure whereas C-Mc reversed this trend and markedly downregulated the production of MMP-1 and MMP-3.

As predicted by the gene enrichment analysis, the MAPK/AP-1 signaling pathway might be regulated by C-Mc, which is closely relevant to the expression of MMPs. To better understand the molecular mechanisms of C-Mc, we examined the MAPK family in NHDFs. Cells treated with UVB radiation showed specific increases in the phosphorylation of p38, ERK, and JNK compared with nonirradiated cells (Figure 7(e)). Treatment with C-Mc suppressed phosphorylation of these MAPKs in a dose-dependent manner. In particular, treatment with 20 *μ*M C-Mc reduced p-p38, p-ERK, and p-JNK expression by 65.3%, 72.1%, and 60.23% inhibition, respectively. We next evaluated the phosphorylation of AP-1 by Western blotting. As shown in Figure 7(f), UVB radiation triggered phosphorylation of both c-Jun and c-Fos proteins. Compared with nonirradiated cells, the p-c-Jun and p-c-Fos levels were 167.15% and 175.77% higher, respectively, in UVB-exposed cells whereas treatment with C-Mc (20 *μ*M) noticeably reversed this effect to levels of 104% and 83%, respectively.

### 3.8. C-Mc Inhibited UVB-Induced Inflammatory Cytokine Secretion and Alleviated NF-*κ*B/I*κ*B-*α* Expression

**Figure 8:**
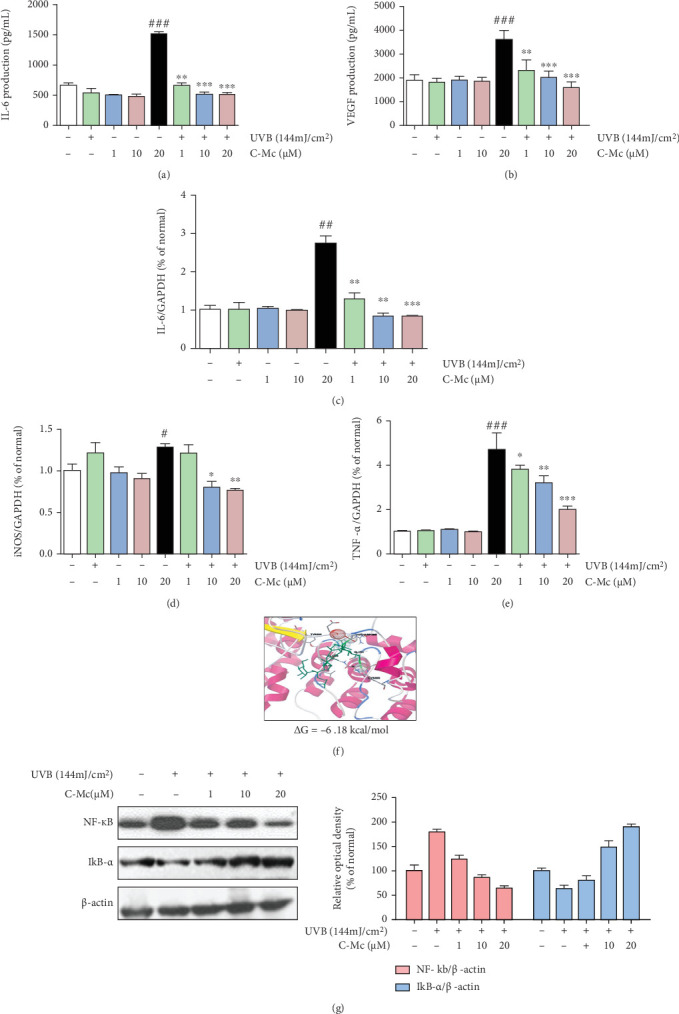
Ginsenoside C-Mc inhibited UVB-induced inflammatory cytokine secretion and alleviated UVB-induced NF-*κ*B/I*κ*B-*α* expression. Production of (a) IL-6 and (b) VEGF under non-UVB irradiation and UVB-irradiated conditions; (c) IL-6, (d) iNOS, and (e) TNF-*α* mRNA expression. An equimolar quantity of mRNA was quantified compared to GAPDH. Cells were incubated in absence or presence of ginsenoside C-Mc at the present concentration after exposure to UVB radiation (144 mJ/cm^2^). (f) Molecular docking simulation for C-Mc binding to NF-*κ*B. (g) The protein levels of NF-*κ*B and I*κ*B-*α* detected by Western blot. Values shown are the mean ± SD. # and ∗ indicate significant differences from the nonirradiated control and UVB-irradiated control groups. ^#^*p* < 0.05, ^##^*p* < 0.01, and ^###^*p* < 0.001 contrast with the nonirradiated control. ⁣^∗^*p* < 0.05, ⁣^∗∗^*p* < 0.01, and ⁣^∗∗∗^*p* < 0.001 contrast with the UVB-irradiated control.

To validate the effects of C-Mc on inflammation functional module, we tested the changes of inflammatory cytokine secretion in UVB-irradiated NHDFs. As presented in Figures 8(a) and 8(b), exposure of NHDFs to UVB irradiation (144 mJ/cm^2^) promoted secretion of IL-6 and VEGF, whereas treatment with C-Mc from 1 to 20 *μ*M significantly blocked IL-6 and VEGF production. Ginsenoside C-Mc quenched decreased VEGF secretion by 52.7%.

We performed q-PCR to assess the effects of C-Mc on iNOS, TNF-*α*, and IL-6 expression in NHDFs. To quantify the results, the ratio of iNOS/GAPDH, TNF-*α*/GAPDH, and IL-6/GAPDH in non-UVB irradiated cells was set to 1.0, according to the signal intensity. In the UVB-exposed group, the mRNA levels of iNOS, TNF-*α*, and IL-6 obviously increased as expected, and this trend was dramatically reversed after C-Mc treatment (Figures 8(c)–8(e)). After treatment with 20 *μ*M C-Mc, expression of the proinflammatory cytokines iNOS, IL-6, and TNF-*α* was downregulated by 61.22%, 60.97%, and 59.88%, respectively.

System pharmacology analysis indicated the important role of C-Mc in the modulation of NF-*κ*B/I*κ*B-*α* pathway. Besides, molecular docking simulation also showed that C-Mc has high affinity to NF-*κ*B with relatively low binding energy (Δ*G*) of -6.18 kcal/mol, indicating that it may directly involve in the regulation of related proteins (Figure 8(f) and Supplementary Table S2). To verify these hypotheses, we evaluated the expression of NF-*κ*B and I*κ*B-*α* by Western blot analysis. Compared with the UVB-exposed control, ginsenoside C-Mc inhibited NF-*κ*B expression in a dose-dependent manner and markedly increased I*κ*B-*α* protein expression (Figure 8(g)).

### 3.9. C-Mc Increased in TGF-*β*1 and Procollagen Type I Secretion and Regulated TGF-*β*/Smad Pathway in UVB-Irradiated NHDFs

**Figure 9:**
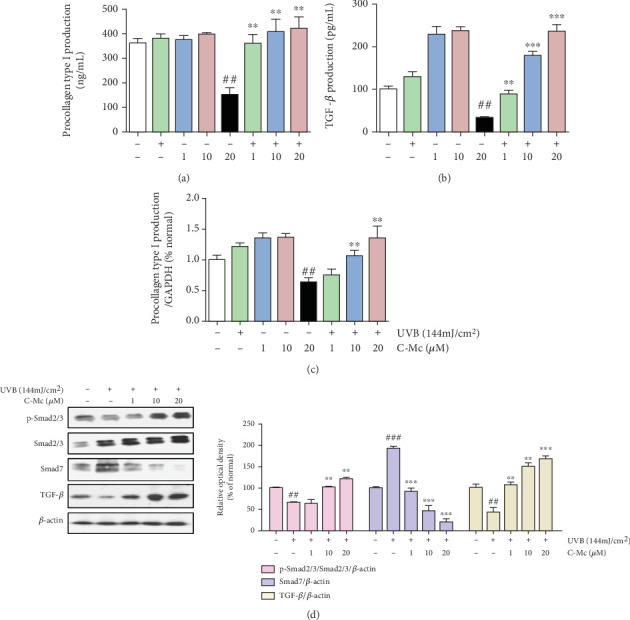
C-Mc increased in TGF-*β*1 and procollagen type I secretion and regulated TGF-*β*/Smad pathway in UVB-irradiated NHDFs. Production of (a) procollagen type I and (b) TGF-*β*1 under non-UVB irradiation and UVB-irradiated conditions. (c) Procollagen type I mRNA expression. An equimolar quantity of mRNA was quantified compared to GAPDH. Cells were incubated in absence or presence of ginsenoside C-Mc at the present concentration after exposure to UVB radiation (144 mJ/cm^2^). (d) The protein levels of Smad2/3, Smad7, and TGF-*β*1 in UVB-irradiated NHDFs were measured by Western blot analysis. The NHDFs were irradiated or nonirradiated with UVB, followed by treated with ginsenoside C-Mc for 1.5 h. The signal intensities for phosphorylation levels of Smad2/3, Smad7, and TGF-*β*1 were tested by Western blotting. Values shown are the mean ± SD. # and ∗ indicate significant differences from the nonirradiated control and UVB-irradiated control groups. ^#^*p* < 0.05, ^##^*p* < 0.01, and ^###^*p* < 0.001 contrast with the nonirradiated control. ⁣^∗^*p* < 0.05, ⁣^∗∗^*p* < 0.01, and ⁣^∗∗∗^*p* < 0.001 contrast with the UVB-irradiated control.

In addition to the inhibition of MMPs and inflammatory cytokine secretion, expediting collagen synthesis is another important functional module of C-Mc predicted by system pharmacology analysis. As shown in Figures 9(a)–9(c), we found that C-Mc treatment affected secretion of TGF-*β*1 and type I procollagen; the levels of type I procollagen and TGF-*β*1 increased by 347% and 294%, respectively, after C-Mc treatment.

The TGF-*β*/Smad signaling pathway plays a crucial role in promoting procollagen synthesis. We examined this pathway in NHDFs after exposure to UVB (144 mJ/cm^2^) and treatment with different concentrations of C-Mc for 1.5 h. As expected, the levels of phosphorylated Smad2/3 and TGF-*β*1 (Figure 9(d)) were drastically upregulated in cells treated with ginsenoside C-Mc, consistent with previous findings.

### 3.10. Effect of C-Mc on the Nrf2/ARE Signaling Pathway Relevant to Oxidative Stress

**Figure 10:**
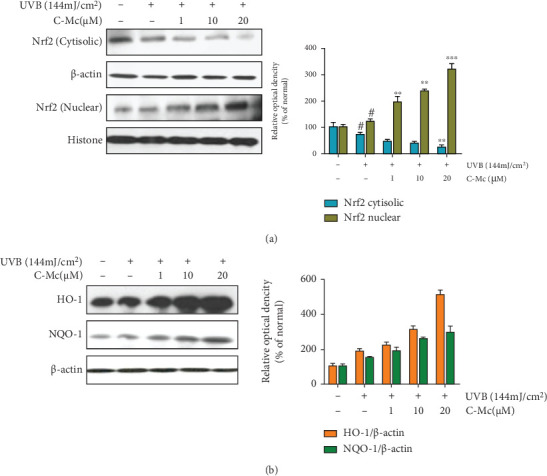
Effects of ginsenoside C-Mc on Nrf2, HO-1, and NQO-1 expression in UVB-irradiated NHDFs. (a) The protein levels of Nrf2 in UVB-irradiated NHDFs were measured by Western blot analysis. The signal intensities for phosphorylation levels of Nrf2. The protein levels of Nrf2 in UVB-irradiated NHDFs were measured by Western blot analysis. (b) The signal intensities for phosphorylation levels of HO-1 and NQO-1. The NHDFs were irradiated or nonirradiated with UVB, followed by treated with ginsenoside C-Mc for 3 h. The results were shown as the mean ± SD of at least three independent experiments. ^#^*p* < 0.05, ^##^*p* < 0.01, and ^###^*p* < 0.001 contrast with the nonirradiated control. ⁣^∗^*p* < 0.05, ⁣^∗∗^*p* < 0.01, and ⁣^∗∗∗^*p* < 0.001 contrast with the UVB-irradiated control.

As the above biological process enrichment and antioxidation experiment results shown, C-Mc possesses significant antioxidant ability against UVB-induced ROS production. To elucidate the detailed mechanism of C-Mc, we further validate the action of C-Mc towards the Nrf2/ARE signaling pathway. Nrf2/ARE signaling is the major regulatory pathway responsible for oxidative stress and further suppresses activation of the NF-*κ*B pathway [55]. We evaluated the level of cytosolic and nuclear Nrf2, NQO-1, and HO-1. After UVB stimulation, C-Mc enhanced aggregation of nuclear Nrf2 proteins, which might imply cellular self-preservation (Figure 10(a)). Simultaneously, treatment with C-Mc dramatically increased NQO-1 expression in UVB-exposed NHDFs in a dose-dependent manner, with 67.35% and 102.14% increases for 20 *μ*M C-Mc (Figure 10(b)).

## 4. Discussion

Interest and research on botanicals as the most promising treatments for skin photoaging have increased in recent years [56]. Because natural active compounds are continually confirmed to have fewer side effects than chemical-based compounds, increasing numbers of researchers have attempted to extract functionalized materials from naturally occurring substances such as flowers, seeds, roots, and essential oils for use in cosmetics [57]. Medicinal Panax herbs especially *Panax quinquefolius* L. have been identified as a plant with wide-ranging effects and have been used as a powerful skin antiaging agent [10]. In particular, many studies have assessed for skin antiaging activity of ginsenosides, the principal active components of American ginseng [58]. The major ginsenosides, Rd, Rg1, and Rc, are glycosylated ginsenosides that are poorly absorbed into the gastrointestinal tract. In contrast, minor ginsenosides present in deglycosylated states are relatively easily absorbed into the bloodstream but account for less than 1% of total ginseng [59]. Owing to the difficulty of extracting and separating of the minor ginsenosides, the identification of the activity of medicament remains a challenge, which hampers their use as drug candidates or cosmetics ingredients.

In this study, we proposed a novel *in silico* framework for the identification of active ginsenosides and exploration of underlying mechanisms against photoaging. The drug virtual screening framework consists of the following steps: (1) preliminary identification based on shortest distance calculation in the chemical space, (2) candidates prioritizing through pairwise chemical structure similarity analysis, and (3) drug-likeness evaluation and ADMET prediction for the candidates (Supplementary Figure S1). We highlighted C-Mc (20-O-[*α*-L-arabinofuranosyl-(1→6)-*β*-D-glucopyranosyl]-20(S)-protopanaxdiol), a rare minor ginsenoside, as one of the most promising candidates for the prevention and treatment of photoaging. We further obtained the minor ginsenoside C-Mc through the Rc→C-Mc1→C-Mc→C-K biotransformation pathway and validated its pharmacologic effects of antiphotoaging in UVB-irradiated NHDFs.

**Figure 11:**
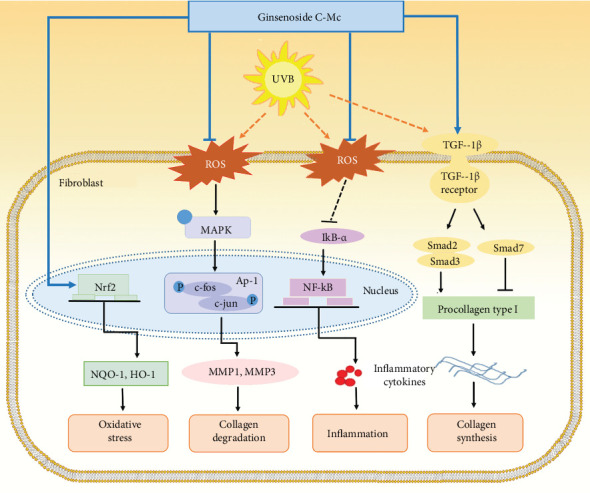
Schematic summary illustrating amelioration of ginsenoside C-Mc on photoaging in UVB-irradiated NHDFs via modulating oxidation stress, inflammation, matrix metalloproteinase secretion, collagen degradation, and synthesis.

System pharmacology analysis was conducted for the exploration of the molecular mechanisms of ginsenosides C-Mc against photoaging. We constructed drug-target network of C-Mc, photoaging-related gene network, and skin tissue-specific expression protein network and performed network analysis and gene enrichment. We predicted four major functional modules of C-Mc against photoaging, including inflammation, collagen synthesis and metabolic, secretion of MMPs, and oxidative stress, which are intrinsically connected. *In vitro* experiments confirmed these hypotheses and further elucidated the cytoprotective mechanism of actions for C-Mc, as summarized in Figure 11.

UVB is the primary external factor that induces ROS, which in turn stimulate many cascades and ultimately lead to premature skin senescence [60]. We demonstrated that ginsenoside C-Mc prevented ROS production (Figures 4(b)–4(d)) and decreased MMP secretion (Figures 7(a)–7(d)) caused by UVB irradiation and showed a protective action against photooxidative aging. Furthermore, C-Mc effectively eliminated UVB-exposed ROS generation and upregulated expression of the antioxidant enzymes HO-1 and NQO-1 by facilitating translocation of Nrf2 from the cytoplasm to the nucleus (Figure 10). These data indicate that C-Mc has antioxidant properties by increasing levels of key endogenous cutaneous antioxidant factors.

UV radiation activates an intricate cascade of biochemical reactions in human skin. UV radiation is known to be a strong activator of skin NF-*κ*B via suppression of I*κ*B-*α* protein synthesis [61]. Expression of NF-*κ*B stimulates the release of proinflammatory cytokines such as TNF-*α*, iNOS, and IL-6 [62]. Previous research showed that TNF-*α* and IL-6 inhibit the production of collagen, and iNOS dramatically suppresses ECM synthesis by reducing the expression of type I collagen [63]. Consistent with previously published studies, we showed that C-Mc significantly inhibited the expression of IL-6, iNOS, and TNF-*α* (Figures 8(a) and 8(c)–8(e)), increased TGF-*β*1 secretion (Figures 9(b) and 9(d)), and upregulated procollagen type I (Figures 9(a) and 9(c)) expression to facilitate collagen synthesis in a concentration-dependent manner. In addition, molecular dynamics simulation manifested C-Mc has potentially therapeutic photoaging effect by suppressing MAPK, NF-*κ*B, IL-6, and TNF-*α* (Supplementary Figure S2 and Table S2). The MAPK/NF-*κ*B pathway stimulates expression of inflammation-related genes and is associated with the MMP overexpression induced by UVB irradiation, especially MMP-1 [64]. Use of the MAPK inhibitors PD98950 (an ERK inhibitor) and SP600125 (a JNK inhibitor) revealed relationships between MMP-1, IL-6, and the MAPK pathway in the antiaging mechanism of C-Mc (Supplementary Figure S5). As expected, the MAPK inhibitors PD98950 and SP600125 decreased expression of MMP-1 and IL-6, indicating that ginsenoside C-Mc inhibits expression of these proteins through regulation of the MAPK signaling pathway. Moreover, C-Mc scavenged ROS (Figures 4(b)–4(d)), as illustrated by reduced LDH and increased intracellular GSH expression levels in UVB-exposed NHDFs (Figures 4(f) and 4(g)). This finding suggests that the antiphotoaging effects of C-Mc are due to activation or induction of antioxidant constituents that can reduce the overproduction of ROS. In general, we hypothesize that C-Mc is an excellent natural anti-inflammatory and antioxidant product for photodamaged skin.

As the precursor of collagen type I, procollagen type I is important for maintaining skin flexibility and resilience [65]. Repetitive or acute exposure to UV irradiation stimulates excessive MMP-1 secretion, which a significant cause of loss of type I collagen in the dermal ECM and alters the physical properties of skin, leading to primary skin aging [66]. UV radiation inhibits procollagen type I synthesis through the TGF-*β*/Smad pathway [67]. Consistent with these findings, C-Mc showed bioactivity associated with upregulation of TGF-*β*1, enhanced phosphorylation of Smad2/3 expression, and remarkably reduced Smad7 expression level in UVB-exposed NHDFs (Figure 9(d)). Therefore, we suggest that regulation of factors associated with the TGF-*β*/Smad pathway might effectively protect cells against UVB-induced photodamage.

Dermatology studies show that VEGF plays a critical role in pathological mechanisms of skin psoriasis and carcinoma [68]. Moreover, VEGF overexpression enhances sensitivity to UVB irradiation while accelerated inflammation expedites photoaging [69]. Broadly speaking, weaken VEGF secretion could be an effective approach to apply to the prevention or treatment of UVB-induced photoaging. Our findings indicated that elevated VEGF secretion resulting from UVB-exposed is conspicuously lowered by treated ginsenoside C-Mc (Figure 8(b)). Hence, mechanisms of UVB-exposed alter in VEGF expression deserved for further investigation in the future.

Overall, the results of this study indicated that the *in silico* approaches proposed here may serve as a novel and effective strategy to accelerate the discovery of antiphotoaging agent from ginsenosides. Yet several shortcomings of the presented study should be acknowledged. Firstly, this study predicted that three rare minor ginsenosides (C-Mc, Mx, and F2) may possess high potential as antiphotoaging agents. Although C-Mc has shown significant antioxidant and cytoprotective activity against UVB-induced photodamage in human dermal fibroblasts, the remaining ginsenosides (Mx and F2) deserved to be further validated by wet-lab assays in the future. Secondly, this study merely preliminary investigated the antiphotoaging effects and mechanisms at the cellular level; further in-depth experiments should be conducted in mice, artificial skin, and human skin to explore the safety and effectiveness of ginsenoside C-Mc as orally or tropically agent.

## 5. Conclusion

This study presented a novel and useful *in silico* drug discovery strategy and identified the rare minor ginsenoside C-Mc as a promising antiphotoaging compound from over 80 ginsenosides. In combination with system pharmacology-based prediction and *in vitro* validation, we found that C-Mc suppressed MMP production via regulating the MAPK/AP-1/NF-*κ*B pathway as well as expedited collagen synthesis via the TGF-*β*/Smad pathway. In addition, C-Mc enhanced the expression of Nrf2/ARE to hold a balance of endogenous oxidation. We believe that C-Mc will prove to be a useful cosmetic agent and drug candidate, particularly for skin care, and might be used to reduce UVB-induced skin photodamage.

## Figures and Tables

**Table 1:** Drug-likeness evaluation and ADMET screening for the 10 predicted antiphotoaging ginsenosides.

Drug	Drug-likeness	PAINS	*F*_20%_	PPB	AOT	Skin sensitization
Ginsenoside XVII	0.848	0	0.986	72.66%	0	0.005
Ginsenoside C-Mx1	0.854	0	0.981	75.10%	0	0.008
NSC308876	0.856	0	0.986	72.90%	0	0.005
*Ginsenoside F2*	0.846	0	0.67	83.91%	0	0.015
Ginsenoside Rs2	0.812	0	0.996	60.39%	0	0.002
Ginsenoside Rs1	0.804	0	0.995	60.82%	0	0.002
*Ginsenoside C-Mc*	0.818	0	0.608	86.59%	0	0.034
Ginsenoside LXXV	0.818	0	0.753	83.36%	0	0.023
*Ginsenoside Mx*	0.764	0	0.655	86.74%	0	0.032
(20R)-Ginsenoside Rh2	0.772	0	0.076	94.05%	0	0.038

Note: PAINS: pan assay interference compounds; *F*_20%_: human oral bioavailability 20%; PPB: plasma protein binding; AOT: rat acute oral toxicity. The value of drug-likeness refers to the probability of being positive. The value of PAINS refers to the number of alert substructures; PPB with predicted value < 90% is considered proper. The values of *F*_20%_, AOT, and skin sensitization are the probabilities of being *F*_20%+_ (bioavailability < 20%), toxicity (<500 mg/kg), and sensitizer, while probability higher than 0.7 represents a poor result according to the explanation of ADMETlab 2.0 [34].

## Data Availability

The original contributions presented in the study are included in the article/supplementary material; further inquiries can be directed to the corresponding authors upon reasonable request.

## Supplementary Materials

Supplementary Materials Figure S1: overall workflow of this study. Figure S2: molecular docking mode of the complex 3D structure. Figure S3: chromatographic analysis of ginsenoside C-Mc. Figure S4: cell viability of ginsenoside C-Mc. Figure S5: effects of MAPK inhibitors on UVB-exposed MAPK activation and MMP-1, IL-6 secretion in UVB-irradiated NHDFs. Table S1: information, structures, and antiphotoaging reference of ginsenosides collecting from literatures and PubChem database. Table S2: binding energy and hydrogen bonding interactions between atomic groups of receptor and ligand. Table S3: drug-target network of ginsenoside C-Mc. Table S4: list of the 124 skin photoaging-related genes. Table S5: the skin tissue-specific expression protein network containing 907 proteins. Table S6: real-time PCR primers. Table S7: the 22 ginsenosides with significant shortest distance (*D*_min_ < 0.2) to the set of known antiphotoaging ginsenosides.
