# Supplementary material for: Chemical Distance Measurement and System Pharmacology Approach Uncover the Novel Protective Effects of Biotransformed Ginsenoside C-Mc against UVB-Irradiated Photoaging
Source: Oxid Med Cell Longev. 2022 Feb 9;2022:4691576. doi: 10.1155/2022/4691576 (PMC8850047; doi:10.1155/2022/4691576)
Supplement: Supplementary Materials — Figure S1: overall workflow of this study. Figure S2: molecular docking mode of the complex 3D structure. Figure S3: chromatographic analysis of ginsenoside C-Mc. Figure S4: cell viability of ginsenoside C-Mc. Figure S5: effects of MAPK inhibitors on UVB-exposed MAPK activation and MMP-1, IL-6 secretion in UVB-irradiated NHDFs. Table S1: information, structures, and antiphotoaging reference of ginsenosides collecting from literatures and PubChem database. Table S2: binding energy and hydrogen bonding interactions between atomic groups of receptor and ligand. Table S3: drug-target network of ginsenoside C-Mc. Table S4: list of the 124 skin photoaging-related genes. Table S5: the skin tissue-specific expression protein network containing 907 proteins. Table S6: real-time PCR primers. Table S7: the 22 ginsenosides with significant shortest distance (Dmin < 0.2) to the set of known antiphotoaging ginsenosides. [file 4691576.f1.pdf]

Supplementary materials

Chemical Distance Measurement and Systems Pharmacology Approach Uncover the Novel Protective Effects of Bio-transformed Ginsenoside C-Mc Against UVB-Irradiated Photoaging

Xiao-yi Liu[1,2,3], Hui Li[1], Eunson Hwang[3], Bom Park[3], Yong-kun Xiao[3], Senmiao Liu[2], Jiansong Fang[4], Yeon-Ju Kim[3]*, Tae-Hoo Yi[3]*, Chuipu Cai[2,4]*

[1]School of Basic Medical Sciences, Guangzhou University of Chinese Medicine, Guangzhou, China.

[2]Division of Data Intelligence, Department of Computer Science, Key Laboratory of Intelligent Manufacturing Technology of Ministry of Education, College of Engineering, Shantou University, Shantou, China.

[3]Department of Oriental Medicinal Biotechnology, College of Life Sciences, Kyung Hee University, Republic of Korea.

[4]Science and Technology Innovation Center, Guangzhou University of Chinese Medicine, Guangzhou, China.

***Correspondence:**

Chuipu Cai, chpcai@stu.edu.cn;

Yeon-Ju Kim, yeonjukim@khu.ac.kr;

Tae-Hoo Yi, drhoo@khu.ac.kr.

Figures

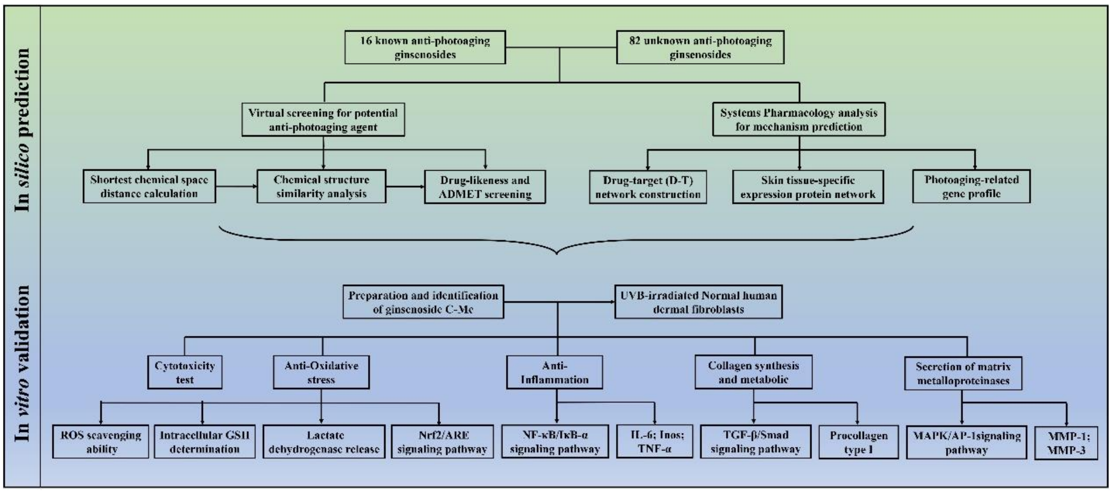

Figure S1. Overall workflow of this study.

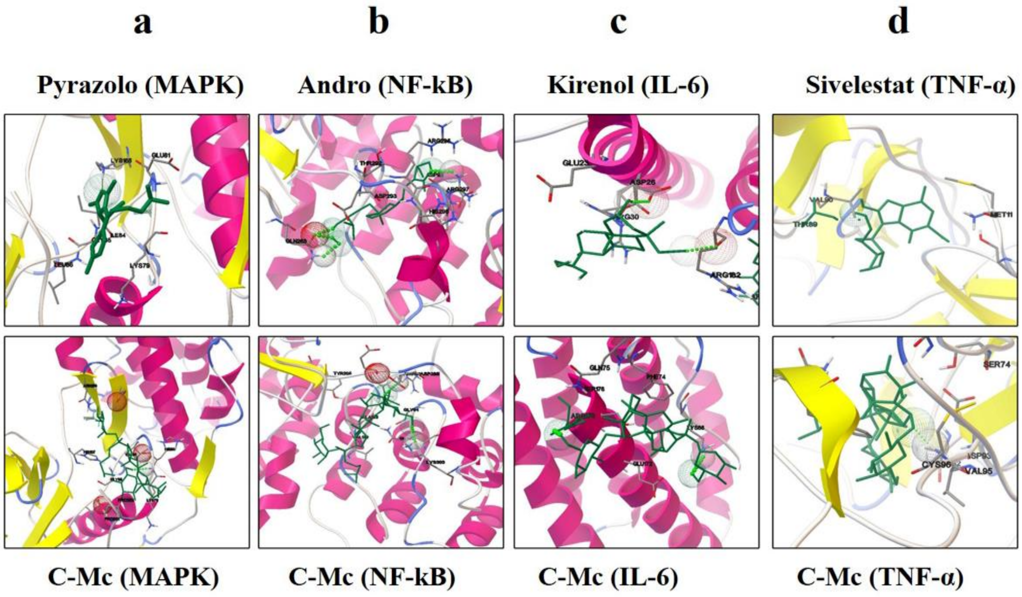

Figure S2. Molecular docking mode of the complex 3D structure. (a) Docking interaction of MAPK with Pyrazolo and C-Mc. **(b)** Docking interaction of NF-κB with Andro and C-Mc. **(c)** Docking interaction of IL-6 with Kirenol and C-Mc. **(d)** Docking interaction of TNF-α with Sivelestat and C-Mc. The 3D structures were optimized in ChemBioOffice 2010 tool package (PerkinElmer Inc.) and the molecular docking simulation was carried by Autodock v4.2 according to the available standard autodock protocol. The reaction conditions were optimized using the control ligand molecules and the lowest binding energy of the target was calculated using Lamarckian Genetic Algorithm (LGA).

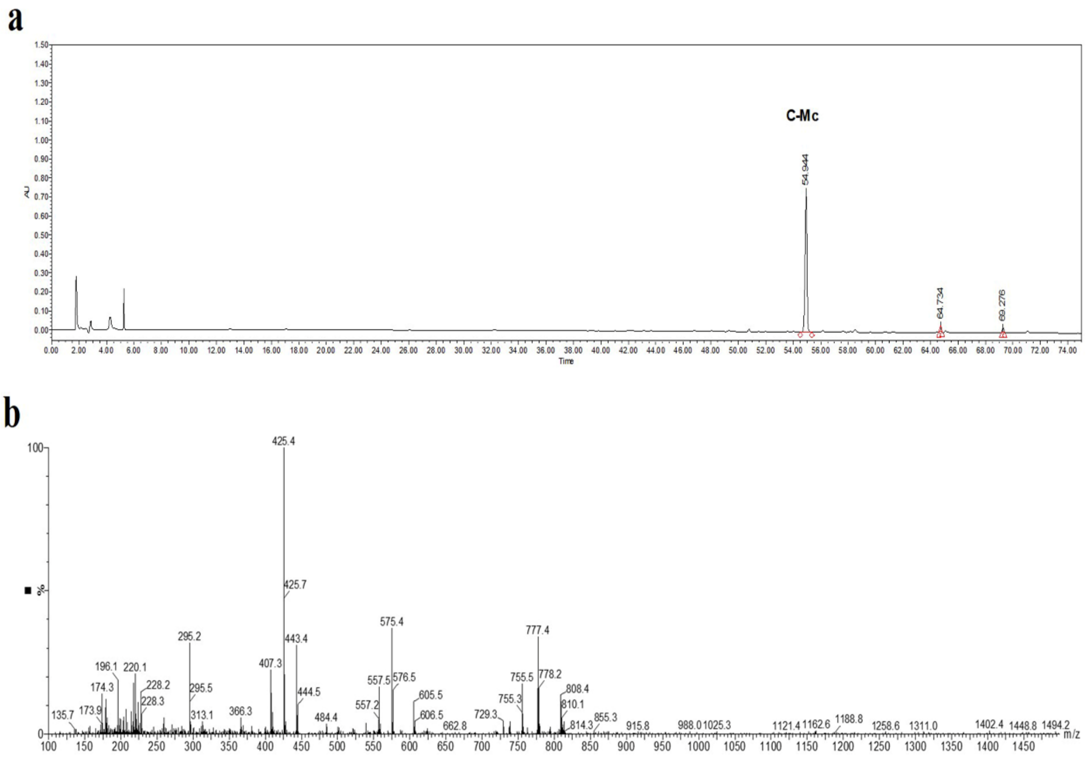

Figure S3. Chromatographic analysis of ginsneoside C-Mc. (a) HPLC analysis of ginsneoside C-Mc; **(b)** Mass chromatograms analysis of ginsneoside C-Mc.

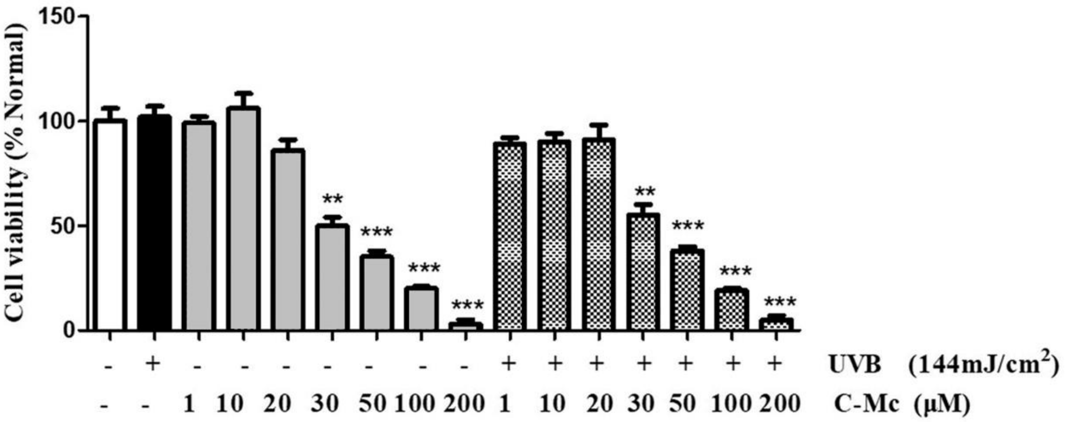

Figure S4. Cell viability of ginsenoside C-Mc. NHDFs were irradiated or non-irradiated with 144 mJ/cm^2 UVB, followed by treatment with the indicated of ginsenoside C-Mc (1, 10, 20, 30, 50, 100 and 200 μM). All data are shown as the mean ± SD of three independent experiments. * indicate significant differences from the non-irradiated control and UVB-irradiated control groups. * $p < 0.05$, ** $p <0.01$ and *** $p < 0.001$ contrast with the UVB-irradiated control.

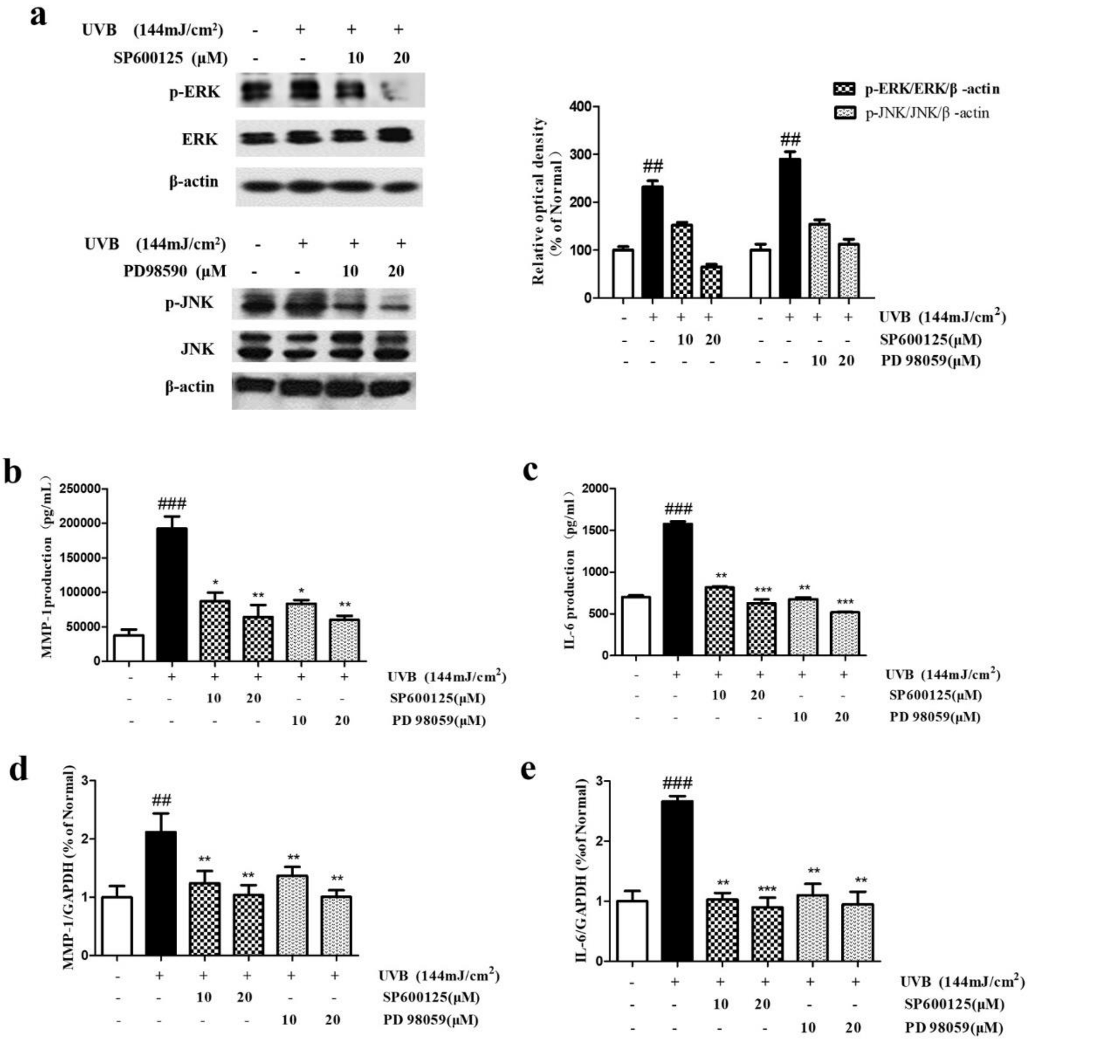

Figure S5. Effects of MAPK inhibitors on UVB-exposed MAPK activation and MMP-1, IL-6 secretion in UVB-irradiated NHDFs. NHDFs were pretreated with indicated concentrations of inhibitors (PD98590 and SP600125) for 2h, followed by UVB (144 mJ/cm^2) irradiated, the cells were then incubated for 30min or 1h. **(a)** The protein levels of ERK and JNK in UVB-irradiated NHDFs were measured by Western blot analysis. The signal intensities for phosphorylation levels of ERK and JNK; Secreted **(b)** MMP-1, **(c)** IL-6 72h after irradiation were determined by ELISA kit. The mRNA expression of (**d**) MMP-1 and **(e)** IL-6 were measured by Real-Time PCR. The results are shown as the mean ± SD of three independent experiments performed in triplicate. Number signs indicate a highly significant difference from the control cells. #$p < 0.05$, ## $p < 0.01$. Asterisks indicate a highly significant difference from the UVB-irradiated cells. * $p < 0.05$, ** $p < 0.01$.

Tables

Table S1. Information, structures and anti-photoaging reference of ginsenosides collecting from literatures and PubChem database.

Pubchem CID	Class	Name	Inchikey	Source	PMID
12855889	Known anti-photoaging agent	Ginsenoside Rc	AGBCLJAHARWNLA-DQUQINEDSA-N	American ginseng;Panax ginseng;Panax notoginseng;Panacis Quinquefolii Radix	28713942
119307	Known anti-photoaging agent	Ginsenoside Rh2	CKUVNOCSBYYHIS-IRFFNABBSA-N	American ginseng;Panax ginseng	25116621
9918693	Known anti-photoaging agent	Ginsenoside Rg3	GZYPWOGIYAIIPV-JBDTYSNRSA-N	American ginseng;Panax ginseng;Panax japonicus var. major	25056231
11815492	Known anti-photoaging agent	Ginsenoside Ro	JDCPEKQWFDWQLI-LUQKBWBOSA-N	American ginseng;Panax ginseng C.A. Meyer	26214051
441921	Known anti-photoaging agent	Ginsenoside Re	NFZYDZXHKFHPGA-QQHDHSITSA-M	American ginseng;Panax ginseng;Panacis Quinquefolii Radix	29755556
12901617	Known anti-photoaging agent	Ginsenoside Rg3(S)	NODILNFGTFIURN-GZPRDHCNSA-N	Panax ginseng;Panax japonicus var. major	28793178
12912363	Known anti-photoaging agent	Ginsenoside Rb3	NODILNFGTFIURN-USYOXQFSSA-N	Panax notoginseng;Panacis Quinquefolii Radix	26287932
122228242	Known anti-photoaging agent	Ginsenoside R7	NTYAVUNEPXGZQJ-LUSMHSHMSA-M	Panax vietnamensis	31111375

N/A	Known anti-photoaging agent	Ginsenoside C-Mx	PWAOOJDMFUQOKB-WCZZMFLVSA-N	Panax notoginseng	29779217
441923	Known anti-photoaging agent	Ginsenoside Rg1	RWXIFXNRCLMQCD-JBVRGBGGSA-N	American ginseng;Panax ginseng;Panax japonicus var. major	26011399
6917976	Known anti-photoaging agent	Ginsenoside Rb2	RWXIFXNRCLMQCD-UHFFFAOYSA-N	American ginseng;Panax ginseng;Panax notoginseng;Panacis Quinquefolii Radix	25774540
21599924	Known anti-photoaging agent	Ginsenoside Rg2	SHCBCKBYTHZQGZ-DLHMIPLTSA-N	American ginseng;Panax ginseng;Panacis Quinquefolii Radix	27816645
118987129	Known anti-photoaging agent	Ginsenoside Rb(1)	VRWPEBVWQIPLBM-AZIBQOHNSA-N	Panax ginseng;American ginseng	19041641
9847853	Known anti-photoaging agent	Protopanaxatriol	YNBYFOIDLBTOMW-JCZNUTPYSA-N	Panax ginseng	33841016
9898279	Known anti-photoaging agent	Ginsenoside Rb1	YNBYFOIDLBTOMW-QHNUHGIDSA-N	American ginseng;Panax ginseng;Panacis Quinquefolii Radix;Panax japonicus var. major	26189299
21672570	Known anti-photoaging agent	Ginsenoside C-Y	YURJSTAIMNSZAE-HHNZYBFYSA-N	Ginsenoside C-Y	31074886

75412551	Unknown anti-photoaging agent	20R-Ginsenoside Rg2	AGBCLJAHARWNLA-UHFFFAOYSA-N	Panax ginseng Meyer	N/A
85193319	Unknown anti-photoaging agent	Ginsenoside Rh7	ARPGURKWJGBPTN-UHFFFAOYSA-N	Panax ginseng	N/A
76602687	Unknown anti-photoaging agent	Ginsenoside Ia	AVTXSAWPGCSYFO-UHFFFAOYSA-N	Panax ginseng	N/A
75412555	Unknown anti-photoaging agent	Ginsenoside Rk3	AVXFIVJSCUOFNT-UHFFFAOYSA-N	Ginseng;Panax notoginseng	N/A
44584745	Unknown anti-photoaging agent	Ginsenoside R10	CFOKFXFKMQABRM-UHFFFAOYSA-N	Panax quinquefolium L	N/A
9896928	Unknown anti-photoaging agent	Ginsenoside Mc	CJFGBCWGOQRURQ-UHFFFAOYSA-N	Panax notoginseng	N/A
14081290	Unknown anti-photoaging agent	(20R)-Ginsenoside Rh2	CKUVNOCSBYYHIS-UHFFFAOYSA-N	Panax ginseng;Panax ginseng Meyer	N/A
13386145	Unknown anti-photoaging agent	Chikusetsusaponin Ia	DGSOBIYFLJXVQZ-UHFFFAOYSA-N	Panax japonicus C. A. Meyer	N/A
24721561	Unknown anti-photoaging agent	Ginsenoside-Rd	FBFMBWCLBGQEBU-UHFFFAOYSA-N	American ginseng;Panax ginseng; Panax japonicus var. major	N/A
21599925	Unknown anti-photoaging agent	Notoginsenoside R2	FNIRVWPHRMMRQI-UHFFFAOYSA-N	Panax notoginseng	N/A

9852086	Unknown anti-photoaging agent	Ginsenosid C-K	FVIZARNDLVOMSU-UHFFFAOYSA-N	Panax ginseng	N/A
14162969	Unknown anti-photoaging agent	Malonyl ginsenoside Rb2	FWCWPAUCBWOOGG-UHFFFAOYSA-M	Panax ginseng	N/A
71587485	Unknown anti-photoaging agent	Gypenoside III	GZYPWOGIYAIIPV-UHFFFAOYSA-N	Panax ginseng;Panax japonicus var.major	N/A
85044013	Unknown anti-photoaging agent	Ginsenoside Rs1	HAJTYGKLQDXTPO-UHFFFAOYSA-N	Panax japonicus var. major	N/A
46887678	Unknown anti-photoaging agent	Ginsenoside F3	HJRVLGWTJSLQIG-UHFFFAOYSA-N	Panax ginseng	N/A
118753434	Unknown anti-photoaging agent	20(S)-Ginsenoside RH2 metabolite M3-6	HOAFPPYAYXARJZ-UHFFFAOYSA-N	Panax ginseng	N/A
122173224	Unknown anti-photoaging agent	(R) Ginsenoside Rh2	HPKGRCABTFZLLX-UHFFFAOYSA-N	Panax ginseng	N/A
90478300	Unknown anti-photoaging agent	Ginsenoside-M6A	IAEZLXLDZBZQPU-UHFFFAOYSA-N	Panax ginseng	N/A
328775	Unknown anti-photoaging agent	Ginsenoside A1	JBGYSAVRIDZNKA-UHFFFAOYSA-N	Panax quinquefolium L.	N/A
100018	Unknown anti-photoaging agent	Panaxoside RC	JDCPEKQWFDWQLI-UHFFFAOYSA-N	Panax ginseng	N/A

131752646	Unknown anti-photoaging agent	Ginsenoside Rh6	JFTBERIHMIFXML-UHFFFAOYSA-N	Hydroponic Panax ginseng	N/A
56949839	Unknown anti-photoaging agent	12,6'-Dioctanoyl Ginsenoside Rh2	KCPBBZLPOURRDV-UHFFFAOYSA-N	Panax species	N/A
118753436	Unknown anti-photoaging agent	20(S)-Ginsenoside RH2 metabolite M3-10	KFHAXWKYIZJFQE-UHFFFAOYSA-N	Panax ginseng	N/A
10629247	Unknown anti-photoaging agent	Vina-ginsenoside R25	KJZQRDAZSWKILI-UHFFFAOYSA-N	Panax vietnamensis	N/A
131801343	Unknown anti-photoaging agent	Ginsenoside-Rg6	KSQILLJRLXANDD-UHFFFAOYSA-N	Steamed notoginseng	N/A
100941542	Unknown anti-photoaging agent	Ginsenoside Ra1	KVMXBSSOCCPAOR-UHFFFAOYSA-N	Panax notoginseng	N/A
124202620	Unknown anti-photoaging agent	Ginsenoside Rk1, analytical standard	KWDWBAISZWOAHD-UHFFFAOYSA-N	Heat-processed Panax ginseng Meyer	N/A
46887590	Unknown anti-photoaging agent	Ginsenoside F5	KWRQPASKWCJCPI-UHFFFAOYSA-N	Panax ginseng	N/A
73981591	Unknown anti-photoaging agent	Ginsenoside Ra0	LDIAQNKCRRXZCD-UHFFFAOYSA-N	Panacis Quinquefolii Radix	N/A

90658963	Unknown anti-photoaging agent	Netoginsenoside R1	LLPWNQMSUYAGQI-UHFFFAOYSA-N	Panax notoginseng	N/A
44592857	Unknown anti-photoaging agent	Vinaginsenoside R6	MGROSLQKCJXSBT-UHFFFAOYSA-N	Panax vietnamensis HA et GRUSHV.	N/A
90657714	Unknown anti-photoaging agent	Notoginsenoside Fe	MYBAONSAUGZRAX-UHFFFAOYSA-N	Panax notoginseng	N/A
92043620	Unknown anti-photoaging agent	Vinaginsenoside R3	NJPDRQDELKMUTI-UHFFFAOYSA-N	Panax quinquefolium L.	N/A
11550001	Unknown anti-photoaging agent	Ginsenoside-Rg5	NJUXRKMKOFXMRX-UHFFFAOYSA-N	Fine black ginseng	N/A
73598	Unknown anti-photoaging agent	Ginsenoside C2	NODILNFGTFIURN-UHFFFAOYSA-N	Panax ginseng;Panax notoginseng;Panacis Quinquefolii Radix	N/A
3086263	Unknown anti-photoaging agent	Malonyl-ginsenoside Rb1	NTYAVUNEPXGZQJ-UHFFFAOYSA-M	Panax japonicus var	N/A
102294900	Unknown anti-photoaging agent	Ginsenoside Km	NUJWGKNPSIVUOO-UHFFFAOYSA-N	Panax ginseng	N/A
3083376	Unknown anti-photoaging agent	Pseudo-ginsenoside-RI3	ONZJEHSXUQSQCU-UHFFFAOYSA-M	Panax pseudo-ginseng	N/A
14162967	Unknown anti-photoaging agent	Malonyl ginsenoside Rd	OSXWNRAKZUNVDR-UHFFFAOYSA-M	Mountain cultivated ginseng (MCG)	N/A
21599928	Unknown anti-photoaging agent	Ginsenoside Rh4	OZTXYFOXQFKYRP-UHFFFAOYSA-N	Panax notoginseng	N/A

20839223	Unknown anti-photoaging agent	Ginsenoside Rh3	PHLXREOMFNVWOH-UHFFFAOYSA-N	Panax ginseng	N/A
21633074	Unknown anti-photoaging agent	Pseudo-ginsenoside RT	PSOUXXNNRFNUAY-UHFFFAOYSA-N	Panax vietnamensis	N/A
58774156	Unknown anti-photoaging agent	Ginsenoside B2	PWAOOJDMFUQOKB-UHFFFAOYSA-N	Panax ginseng;Panacis Quinquefolii Radix	N/A
11213350	Unknown anti-photoaging agent	(20S)-Protopanaxadiol	PYXFVCFISTUSOO-UHFFFAOYSA-N	Panax ginseng	N/A
131751696	Unknown anti-photoaging agent	Ginsenoside I	QCCQLRNQOAQMKU-UHFFFAOYSA-N	Korean ginseng (Panax ginseng C.A. Meyer)	N/A
53447401	Unknown anti-photoaging agent	Ginsenoside-F1	QLPKLNOLHUEWSE-UHFFFAOYSA-N	Panax ginseng	N/A
102004835	Unknown anti-photoaging agent	Ginsenoside F4	QOMBXPYXWGTFNR-UHFFFAOYSA-N	Steamed notoginseng	N/A
6441009	Unknown anti-photoaging agent	SCHEMBL15557558	QOVKKEKBURQILF-UHFFFAOYSA-N	Panax ginseng	N/A
73157064	Unknown anti-photoaging agent	Ginsenoside Ra3	QUNSGRLNZDSQJC-UHFFFAOYSA-N	Panax notoginseng	N/A
12855920	Unknown anti-photoaging agent	(20R)-Ginsenoside Rh1	RAQNTCRNSXYLAH-UHFFFAOYSA-N	Panax ginseng	N/A
432447	Unknown anti-photoaging agent	NSC308876	RLDVZILFNVRJTL-UHFFFAOYSA-N	Panax ginseng	N/A

146160065	Unknown anti-photoaging agent	Ginsenoside Mb	RMHYFFDYVDOCSP-UHFFFAOYSA-N	Panax notoginseng	N/A
11468733	Unknown anti-photoaging agent	Protopanaxatriol	SHCBCKBYTHZQGZ-UHFFFAOYSA-N	Ginseng;Notoginseng	N/A
9918692	Unknown anti-photoaging agent	Ginsenoside F2	SWIROVJVGRGSPO-UHFFFAOYSA-N	Panax ginseng Meyer;Panax ginseng	N/A
100937823	Unknown anti-photoaging agent	Ginsenoside Rs3	TUMCLUKPDAUYFA-UHFFFAOYSA-N	Panax ginseng C.A. Meyer;Korean red ginseng	N/A
100941543	Unknown anti-photoaging agent	Ginsenoside Ra2	UEBIBJSWHIZNCA-UHFFFAOYSA-N	Panax notoginseng	N/A
101835368	Unknown anti-photoaging agent	Ginsenoside MRc	UOFHLCPZXZURFL-UHFFFAOYSA-M	American ginseng;White-ginseng;sanchi-ginseng	N/A
5176390	Unknown anti-photoaging agent	Vinaginsenoside R4	UOJAEODBOCLNBU-UHFFFAOYSA-N	Panax ginseng	N/A
44593678	Unknown anti-photoaging agent	Vinaginsenoside R2	URPKGNJHPFKECW-UHFFFAOYSA-N	Panax vietnamensis	N/A
118753486	Unknown anti-photoaging agent	20(S)-Ginsenoside RH2 metabolite M1-1	UTTXMCFEQIEWFA-UHFFFAOYSA-N	Panax ginseng	N/A
131751552	Unknown anti-photoaging agent	Ginsenoside III	UWGVFBVJCVDUPT-UHFFFAOYSA-N	Panax ginseng;Panacis Quinquefolii	N/A

				Radix;Panax japonicus var. major	
441922	Unknown anti-photoaging agent	Panaxoside RF	UZIOUZHBUYLDHW-UHFFFAOYSA-N	Panax ginseng;Panax japonicus var.major	N/A
101717751	Unknown anti-photoaging agent	Ginsenoside II	VFRFTROKXBCTHW-UHFFFAOYSA-N	Panax ginseng	N/A
85245726	Unknown anti-photoaging agent	Ginsenoside Rh8	VGJOYFZLAIERID-UHFFFAOYSA-N	Panax ginseng	N/A
146158775	Unknown anti-photoaging agent	Gypenoside IV	VMFHKVMQSYOBAV-UHFFFAOYSA-N	Panax ginseng	N/A
130009	Unknown anti-photoaging agent	Ginsenoside-La	VOUCMBDNXOKLCQ-UHFFFAOYSA-N	Panax ginseng C. A. Meyer	N/A
181573	Unknown anti-photoaging agent	20(R)-Ginsenodie Rg3	VOWJAFYNABYJKY-UHFFFAOYSA-N	Red ginseng (Panax ginseng C.A Meyer)	N/A
44144330	Unknown anti-photoaging agent	Vinaginsenoside R1	VTZDPCBUMTUOMY-UHFFFAOYSA-N	American ginseng;Panax vietnamensis	N/A
90472238	Unknown anti-photoaging agent	Ginsenoside Rk2	WMGBQZAELMGYNO-UHFFFAOYSA-N	Sun Ginseng	N/A
85237385	Unknown anti-photoaging agent	Ginsenoside Rh5	WTSNEXSNFSFTFK-UHFFFAOYSA-N	Panax ginseng	N/A
9809542	Unknown anti-photoaging agent	Ginsenoside F1	XNGXWSFSJIQMNC-UHFFFAOYSA-N	Korean Panax ginseng Meyer	N/A

101887367	Unknown anti-photoaging agent	Pseudoginsenoside Rh2	YCDZBVXSGVWFFX-UHFFFAOYSA-N	Panax ginseng	N/A
86289140	Unknown anti-photoaging agent	Gypenoside LXXV	YIYRCZFIJNGYOG-UHFFFAOYSA-N	Panax ginseng	N/A
73157065	Unknown anti-photoaging agent	Ginsenoside Rs2	YLMBQJRKOKVUCP-UHFFFAOYSA-N	Panax japonicus var. major	N/A
11331683	Unknown anti-photoaging agent	Ginsenoside Mx	YNBYFOIDLBTOMW-UHFFFAOYSA-N	Panax ginseng	N/A
432116	Unknown anti-photoaging agent	Ginsenoside A2	YURJSTAIMNSZAE-UHFFFAOYSA-N	Panax ginseng;Panax japonicus var. major	N/A
131752701	Unknown anti-photoaging agent	Ginsenoside M7cd	YWQANVSRCZLIRL-UHFFFAOYSA-N	Panax ginseng C.A. Meyer	N/A
100988292	Unknown anti-photoaging agent	20(S)-Ginsenoside RH2 metabolite M1-5	ZIRHYQFOBJNNCZ-UHFFFAOYSA-N	Panax ginseng	N/A
44584555	Unknown anti-photoaging agent	Gynosaponin S	ZRBFCAALKKNCJG-UHFFFAOYSA-N	Panax ginseng	N/A
73758547	Unknown anti-photoaging agent	Notoginsenoside Fd	ZTQSADJAYQOCDD-UHFFFAOYSA-N	Panax notoginseng	N/A
91895489	Unknown anti-photoaging agent	Ginsenoside Rg6	ZVTVWDXRNMHGNY-UHFFFAOYSA-N	Steamed notoginseng	N/A

102021585	Unknown anti-photoaging agent	Ginsenoside Rs5	ZZTHVSDJFCDCGT-UHFFFAOYSA-N	Panax ginseng	N/A

Table S2. Binding energy and Hydrogen bonding interactions between atomic groups of receptor and ligand.

Protein	Drug	Docking Energy	Amino acid involved in interaction	H-bond distance (Å)	Hydrogen bonding interactions	
					Ligand	Protein
MAPK	Pyrazoloamine (Control)	-5.78	LYS165	1.7	O---	HZ3
	Ginsenoside C-Mc	-5.87	HIS80	1.5	CA---	HN
NF-κB	Andrographolide (Control)	-4.71	GLN263	1.2	H---	HE21
			ARG297	1.1	H---	HH21
	Ginsenoside C-Mc	-6.18	ASP293	1.5	CA---	HN
			LYS303	1.6	CA---	HZ2
IL-6	Kirenol (Control)	-4.07	ARG30	2.2	H---	O---
	Ginsenoside C-Mc	-6.46	ARG179	1.5	O---	HH12
			LYS66	1.5	O---	HZ1
TNF-α	Sivelestat (Control)	-2.97	-	-	-	-
	Ginsenoside C-Mc	-4.53	CYS96	1.7	O---	HN

Table S3. Drug-target network of ginsenoside C-Mc.

Gene Entrez ID	Targets (Coding gene offical symbol)	Organism	Source
3569	IL-6	Homo sapiens (Human)	Complex
4314	MMP3	Homo sapiens (Human)	Complex
4318	MMP9	Homo sapiens (Human)	Database
7421	VDR	Homo sapiens (Human)	bSDTNBI
6868	TNF- α	Homo sapiens (Human)	Literature
3091	HIF1A	Homo sapiens (Human)	Complex
4313	MMP2	Homo sapiens (Human)	Complex
3156	HMGCR	Homo sapiens (Human)	bSDTNBI
136	ADORA2B	Homo sapiens (Human)	bSDTNBI
960	CD44	Homo sapiens (Human)	Database
1588	CYP19A1	Homo sapiens (Human)	bSDTNBI
1956	EGFR	Homo sapiens (Human)	Complex

4312	MMP-1	Homo sapiens (Human)	Literature
595	cyclin D1	Homo sapiens (Human)	Literature
162514	TRPV3	Homo sapiens (Human)	bSDTNBI
2261	ACh,acetylcholine	Homo sapiens (Human)	Literature
7124	TNF	Homo sapiens (Human)	Complex
4790	NF-kB	Homo sapiens (Human)	Complex
4780	Nrf2	Homo sapiens (Human)	Complex
5594	MAPK1	Homo sapiens (Human)	Complex
23411	SIRT1	Homo sapiens (Human)	Complex
3551	IKBKB	Homo sapiens (Human)	Complex
207	Akt1	Homo sapiens (Human)	Complex
3586	IL10	Homo sapiens (Human)	Complex
4843	NOS2	Homo sapiens (Human)	Complex
2526	FUT4	Homo sapiens (Human)	Complex
3667	IRS1	Homo sapiens (Human)	Complex
6523	SGLT1	Homo sapiens (Human)	Complex
35671	iNOS,induciblenitricoxidesynthase	Homo sapiens (Human)	Literature
7040	TGF-β	Homo sapiens (Human)	Literature
100682424	HO-1,hemeoxygenase-1	Homo sapiens (Human)	Literature
100329011	VEGF	Homo sapiens (Human)	Literature
12367	caspase-3	Homo sapiens (Human)	Literature
596	bcl-2	Homo sapiens (Human)	Literature
4087	Smad2/3	Homo sapiens (Human)	Literature
39728892	PI3K	Homo sapiens (Human)	Literature
5562	AMPK	Homo sapiens (Human)	Literature
100533320	JNK	Homo sapiens (Human)	Literature
18033	NF-κB	Homo sapiens (Human)	Literature
4513	COX-2,cyclooxygenase-2	Homo sapiens (Human)	Literature
581	bax	Homo sapiens (Human)	Literature
1017	CDK-2	Homo sapiens (Human)	Literature
1019	CDK-4	Homo sapiens (Human)	Literature
3654	IRAK-1	Homo sapiens (Human)	Literature
1728	NQO-1	Homo sapiens (Human)	Literature
692466	ERK	Homo sapiens (Human)	Literature
778927	p-38	Homo sapiens (Human)	Literature
4286	MITF	Homo sapiens (Human)	Literature
4092	Smad7	Homo sapiens (Human)	Literature
683	BST1	Homo sapiens (Human)	Database

952	CD38	Homo sapiens (Human)	Database
4057	LTF	Homo sapiens (Human)	Database
4837	NNMT	Homo sapiens (Human)	Database
7018	TF	Homo sapiens (Human)	Database
10135	NAMPT	Homo sapiens (Human)	Database
22933	SIRT2	Homo sapiens (Human)	Database
23410	SIRT3	Homo sapiens (Human)	Database
80351	TNKS2	Homo sapiens (Human)	Database
128240	APOA1BP	Homo sapiens (Human)	Database
145482	PTGR2	Homo sapiens (Human)	Database
1559	CYP2C9	Homo sapiens (Human)	Database
2353	FOS	Homo sapiens (Human)	Database
820	CAMP	Homo sapiens (Human)	Database
3162	HMOX1	Homo sapiens (Human)	Database
3553	IL1B	Homo sapiens (Human)	Database
5743	PTGS2	Homo sapiens (Human)	Database
6098	ROS1	Homo sapiens (Human)	Database
148327	CREB3L4	Homo sapiens (Human)	Database
1000	CDH2	Homo sapiens (Human)	Database
1051	Cebpb	Homo sapiens (Human)	Database
1432	MAPK14	Homo sapiens (Human)	Database
3576	IL8	Homo sapiens (Human)	Database
4629	MYH11	Homo sapiens (Human)	Database
5970	RELA	Homo sapiens (Human)	Database
6581	SLC22A3	Homo sapiens (Human)	Database
6615	SNAI1	Homo sapiens (Human)	Database
6657	SOX2	Homo sapiens (Human)	Database
6876	TAGLN	Homo sapiens (Human)	Database
7431	VIM	Homo sapiens (Human)	Database
79923	NANOG	Homo sapiens (Human)	Database
4846	NOS3	Homo sapiens (Human)	Database
5747	PTK2	Homo sapiens (Human)	Database
5829	PXN	Homo sapiens (Human)	Database
6714	SRC	Homo sapiens (Human)	Database
39868499	GLP-1	Homo sapiens (Human)	Literature
2308	FoxO1	Homo sapiens (Human)	Literature
12575	MDA	Homo sapiens (Human)	Literature
6647	SOD	Homo sapiens (Human)	Literature

20850	STAT5-PPAR	Homo sapiens (Human)	Literature
875	CBS,cystathionine-β-synthase	Homo sapiens (Human)	Literature
100194964	CGL,cystathionine-γ-lyase	Homo sapiens (Human)	Literature
1103	ChAT,cholineacetyltransferase	Homo sapiens (Human)	Literature
13130	DA,dopamine	Homo sapiens (Human)	Literature
100033958	eNOS,endothelialnitricoxidesynthase	Homo sapiens (Human)	Literature
2700445	GLUT,glucosetransporter	Homo sapiens (Human)	Literature
100304604	IFN,interferon	Homo sapiens (Human)	Literature
16153	IL,interleukin	Homo sapiens (Human)	Literature
58022	caspase-8	Homo sapiens (Human)	Literature
12371	caspase-9	Homo sapiens (Human)	Literature
947215	IAP	Homo sapiens (Human)	Literature
3355109	PARP	Homo sapiens (Human)	Literature
6774	STAT 3	Homo sapiens (Human)	Literature
3099	HK2	Homo sapiens (Human)	Literature
2768677	p53	Homo sapiens (Human)	Literature
9997	cytochrome c	Homo sapiens (Human)	Literature
222236	mitosis-related proteins	Homo sapiens (Human)	Literature
51339	β-catenin	Homo sapiens (Human)	Literature
100641322	Tcf	Homo sapiens (Human)	Literature
2068	DNA-repair proteins	Homo sapiens (Human)	Literature
1947	Eph/ephrin	Homo spaiens (Human)	Literature
142	PARP-1	Homo sapiens (Human)	Literature
5266	PI3	Homo sapiens (Human)	Literature
51191	cyclin E	Homo sapiens (Human)	Literature
26413	ERK	Homo sapiens (Human)	Literature
9606	GCLC	Homo sapiens (Human)	Literature
2730	GClM	Homo sapiens (Human)	Literature
835008	cp1	Homo sapiens (Human)	Literature
63976	Prdm16	Homo sapiens (Human)	Literature
10891	Pgc1α	Homo sapiens (Human)	Literature
1734	Dio2	Homo sapiens (Human)	Literature
1149	Cidea	Homo sapiens (Human)	Literature
8309	Acox2	Homo sapiens (Human)	Literature
467	Atf3	Homo sapiens (Human)	Literature
854403	MCP-1	Homo sapiens (Human)	Literature
104978912	LDL	Homo sapiens (Human)	Literature

47384	SR-A	Homo sapiens (Human)	Literature
373523	P16	Homo sapiens (Human)	Literature
31381	Rb	Homo sapiens (Human)	Literature
34924	CyclinE	Homo sapiens (Human)	Literature
7450832	MAPK	Homo sapiens (Human)	Literature
51478	PRAP	Homo sapiens (Human)	Literature
6524	SLC5A2	Homo sapiens (Human)	bSDTNBI
6462	SHBG	Homo sapiens (Human)	bSDTNBI
4598	MVK	Homo sapiens (Human)	bSDTNBI
134	ADORA1	Homo sapiens (Human)	bSDTNBI
140	ADORA3	Homo sapiens (Human)	bSDTNBI
135	ADORA2A	Homo sapiens (Human)	bSDTNBI
151306	GPBAR1	Homo sapiens (Human)	bSDTNBI
9453	GGPS1	Homo sapiens (Human)	bSDTNBI
2908	NR3C1	Homo sapiens (Human)	bSDTNBI
9934	P2RY14	Homo sapiens (Human)	bSDTNBI
5770	PTPN1	Homo sapiens (Human)	bSDTNBI
6527	SLC5A4	Homo sapiens (Human)	bSDTNBI
3710	ITPR3	Homo sapiens (Human)	bSDTNBI
1880	GPR183	Homo sapiens (Human)	bSDTNBI
1269	CNR2	Homo sapiens (Human)	bSDTNBI
191	AHCY	Homo sapiens (Human)	bSDTNBI
55775	TDP1	Homo sapiens (Human)	bSDTNBI
4986	OPRK1	Homo sapiens (Human)	bSDTNBI
279	AMY2A	Homo sapiens (Human)	bSDTNBI
5031	P2RY6	Homo sapiens (Human)	bSDTNBI
5241	PGR	Homo sapiens (Human)	bSDTNBI
866	SERPINA6	Homo sapiens (Human)	bSDTNBI
4985	OPRD1	Homo sapiens (Human)	bSDTNBI
2099	ESR1	Homo sapiens (Human)	bSDTNBI
4988	OPRM1	Homo sapiens (Human)	bSDTNBI
367	AR	Homo sapiens (Human)	bSDTNBI
8972	MGAM	Homo sapiens (Human)	bSDTNBI
5029	P2RY2	Homo sapiens (Human)	bSDTNBI
276277278	AMY1A; AMY1B; AMY1C	Homo sapiens (Human)	bSDTNBI
246	ALOX15	Homo sapiens (Human)	bSDTNBI
590	BCHE	Homo sapiens (Human)	bSDTNBI
1268	CNR1	Homo sapiens (Human)	bSDTNBI

5030	P2RY4	Homo sapiens (Human)	bSDTNBI
4306	NR3C2	Homo sapiens (Human)	bSDTNBI
2847	MCHR1	Homo sapiens (Human)	bSDTNBI
2222	FDFT1	Homo sapiens (Human)	bSDTNBI
5034	P4HB	Homo sapiens (Human)	bSDTNBI
6530	SLC6A2	Homo sapiens (Human)	bSDTNBI
100	ADA	Homo sapiens (Human)	bSDTNBI
5028	P2RY1	Homo sapiens (Human)	bSDTNBI
132	ADK	Homo sapiens (Human)	bSDTNBI
2100	ESR2	Homo sapiens (Human)	bSDTNBI
239	ALOX12	Homo sapiens (Human)	bSDTNBI
10599	SLCO1B1	Homo sapiens (Human)	bSDTNBI
2224	FDPS	Homo sapiens (Human)	bSDTNBI
2742	GLRA2	Homo sapiens (Human)	bSDTNBI
23566	LPAR3	Homo sapiens (Human)	bSDTNBI
10062	NR1H3	Homo sapiens (Human)	bSDTNBI
6532	SLC6A4	Homo sapiens (Human)	bSDTNBI
3632	INPP5A	Homo sapiens (Human)	bSDTNBI
1585	CYP11B2	Homo sapiens (Human)	bSDTNBI
29881	NPC1L1	Homo sapiens (Human)	bSDTNBI
2539	G6PD	Homo sapiens (Human)	bSDTNBI
4987	OPRL1	Homo sapiens (Human)	bSDTNBI
5578	PRKCA	Homo sapiens (Human)	bSDTNBI
1576	CYP3A4	Homo sapiens (Human)	bSDTNBI
2741	GLRA1	Homo sapiens (Human)	bSDTNBI
6097	RORC	Homo sapiens (Human)	bSDTNBI
3417	IDH1	Homo sapiens (Human)	bSDTNBI
3043	HBB	Homo sapiens (Human)	bSDTNBI
5739	PTGIR	Homo sapiens (Human)	bSDTNBI
5737	PTGFR	Homo sapiens (Human)	bSDTNBI
3251	HPRT1	Homo sapiens (Human)	bSDTNBI
79915	ATAD5	Homo sapiens (Human)	bSDTNBI
7376	NR1H2	Homo sapiens (Human)	bSDTNBI
5733	PTGER3	Homo sapiens (Human)	bSDTNBI
3290	HSD11B1	Homo sapiens (Human)	bSDTNBI
412	STS	Homo sapiens (Human)	bSDTNBI
6531	SLC6A3	Homo sapiens (Human)	bSDTNBI
240	ALOX5	Homo sapiens (Human)	bSDTNBI

3614	IMPDH1	Homo sapiens (Human)	bSDTNBI
6713	SQLE	Homo sapiens (Human)	bSDTNBI
1584	CYP11B1	Homo sapiens (Human)	bSDTNBI
51053	GMNN	Homo sapiens (Human)	bSDTNBI
57121	LPAR5	Homo sapiens (Human)	bSDTNBI
43	ACHE	Homo sapiens (Human)	bSDTNBI
2548	GAA	Homo sapiens (Human)	bSDTNBI
7442	TRPV1	Homo sapiens (Human)	bSDTNBI
1565	CYP2D6	Homo sapiens (Human)	bSDTNBI
4842	NOS1	Homo sapiens (Human)	bSDTNBI
6915	TBXA2R	Homo sapiens (Human)	bSDTNBI
10724	OGA	Homo sapiens (Human)	bSDTNBI
760	CA2	Homo sapiens (Human)	bSDTNBI
5024	P2RX3	Homo sapiens (Human)	bSDTNBI
5444	PON1	Homo sapiens (Human)	bSDTNBI
8856	NR1I2	Homo sapiens (Human)	bSDTNBI
115584	SLC5A11	Homo sapiens (Human)	bSDTNBI
203068	TUBB	Homo sapiens (Human)	bSDTNBI
5335	PLCG1	Homo sapiens (Human)	bSDTNBI
11255	HRH3	Homo sapiens (Human)	bSDTNBI
2840	GPR17	Homo sapiens (Human)	bSDTNBI

Table S4. List of the 124 skin photoaging-related genes (.xlsx).

Gene ID	Symbol	Species	David Gene Name
8530	CST7	Homo sapiens	cystatin F(CST7)
728279	KRTAP2-2	Homo sapiens	keratin associated protein 2-2(KRTAP2-2)
285313	IGSF10	Homo sapiens	immunoglobulin superfamily member 10(IGSF10)
2551	GABPA	Homo sapiens	GA binding protein transcription factor alpha subunit(GABPA)
83902	KRTAP17-1	Homo sapiens	keratin associated protein 17-1(KRTAP17-1)
57864	SLC46A2	Homo sapiens	solute carrier family 46 member 2(SLC46A2)
1459	CSNK2A2	Homo sapiens	casein kinase 2 alpha 2(CSNK2A2)
402415	XKRX	Homo sapiens	XK related, X-linked(XKRX)
81850	KRTAP1-3	Homo sapiens	keratin associated protein 1-3(KRTAP1-3)
81851	KRTAP1-1	Homo sapiens	keratin associated protein 1-1(KRTAP1-1)
911	CD1C	Homo sapiens	CD1c molecule(CD1C)
152330	CNTN4	Homo sapiens	contactin 4(CNTN4)
9355	LHX2	Homo sapiens	LIM homeobox 2(LHX2)

6001	RGS10	Homo sapiens	regulator of G-protein signaling 10(RGS10)
3891	KRT85	Homo sapiens	keratin 85(KRT85)
3892	KRT86	Homo sapiens	keratin 86(KRT86)
1909	EDNRA	Homo sapiens	endothelin receptor type A(EDNRA)
1745	DLX1	Homo sapiens	distal-less homeobox 1(DLX1)
2159	F10	Homo sapiens	coagulation factor X(F10)
125	ADH1B	Homo sapiens	alcohol dehydrogenase 1B (class I), beta polypeptide(ADH1B)
254773	LYG2	Homo sapiens	lysozyme g2(LYG2)
3883	KRT33A	Homo sapiens	keratin 33A(KRT33A)
3884	KRT33B	Homo sapiens	keratin 33B(KRT33B)
6358	CCL14	Homo sapiens	C-C motif chemokine ligand 14(CCL14)
8935	SKAP2	Homo sapiens	src kinase associated phosphoprotein 2(SKAP2)
3885	KRT34	Homo sapiens	keratin 34(KRT34)
5947	RBP1	Homo sapiens	retinol binding protein 1(RBP1)
6252	RTN1	Homo sapiens	reticulon 1(RTN1)
929	CD14	Homo sapiens	CD14 molecule(CD14)
9244	CRLF1	Homo sapiens	cytokine receptor like factor 1(CRLF1)
6256	RXRA	Homo sapiens	retinoid X receptor alpha(RXRA)
7345	UCHL1	Homo sapiens	ubiquitin C-terminal hydrolase L1(UCHL1)
5320	PLA2G2A	Homo sapiens	phospholipase A2 group IIA(PLA2G2A)
5167	ENPP1	Homo sapiens	ectonucleotide pyrophosphatase/phosphodiesterase 1(ENPP1)
25960	ADGRA2	Homo sapiens	adhesion G protein-coupled receptor A2(ADGRA2)
2207	FCER1G	Homo sapiens	Fc fragment of IgE receptor Ig(FCER1G)
448834	KPRP	Homo sapiens	keratinocyte proline rich protein(KPRP)
3937	LCP2	Homo sapiens	lymphocyte cytosolic protein 2(LCP2)
81872	Krtap2-1	Homo sapiens	keratin associated protein 2-1(KRTAP2-1)
83896	KRTAP3-1	Homo sapiens	keratin associated protein 3-1(KRTAP3-1)
8549	LGR5	Homo sapiens	leucine rich repeat containing G protein-coupled receptor 5(LGR5)
51702	PADI3	Homo sapiens	peptidyl arginine deiminase 3(PADI3)
55507	GPRC5D	Homo sapiens	G protein-coupled receptor class C group 5 member D(GPRC5D)
57216	VANGL2	Homo sapiens	VANGL planar cell polarity protein 2
6387	CXCL12	Homo sapiens	C-X-C motif chemokine ligand 12(CXCL12)
6423	SFRP2	Homo sapiens	secreted frizzled related protein 2(SFRP2)
10085	EDIL3	Homo sapiens	EGF like repeats and discoidin domains 3(EDIL3)

23276	KLHL18	Homo sapiens	kelch like family member 18(KLHL18)
7070	THY1	Homo sapiens	Thy-1 cell surface antigen(THY1)
3394	IRF8	Homo sapiens	interferon regulatory factor 8(IRF8)
144110	TMEM86A	Homo sapiens	transmembrane protein 86A(TMEM86A)
2213	Fcgr2b	Homo sapiens	Fc fragment of IgG receptor IIb(FCGR2B)
50861	STMN3	Homo sapiens	stathmin 3(STMN3)
945	CD33	Homo sapiens	CD33 molecule(CD33)
283298	olfml1	Homo sapiens	olfactomedin like 1(OLFML1)
6899	TBX1	Homo sapiens	T-box 1(TBX1)
9807	IP6K1	Homo sapiens	inositol hexakisphosphate kinase 1(IP6K1)
2736	GLI2	Homo sapiens	GLI family zinc finger 2(GLI2)
64386	MMP25	Homo sapiens	MMP25 – matrix metallopeptidase 25
85293	KRTAP3-3	Homo sapiens	keratin associated protein 3-3(KRTAP3-3)
7122	CLDN5	Homo sapiens	claudin 5(CLDN5)
3684	ITGAM	Homo sapiens	integrin subunit alpha M(ITGAM)
1381	CRABP1	Homo sapiens	cellular retinoic acid binding protein 1(CRABP1)
112802	KRT71	Homo sapiens	keratin 71(KRT71)
2624	GATA2	Homo sapiens	GATA binding protein 2(GATA2)
54796	BNC2	Homo sapiens	basonuclin 2(BNC2)
84645	C22orf23	Homo sapiens	chromosome 22 open reading frame 23(C22orf23)
84000	TMPRSS13	Homo sapiens	transmembrane protease, serine 13(TMPRSS13)
713	C1QB	Homo sapiens	complement C1q B chain(C1QB)
3953	LEPR	Homo sapiens	leptin receptor(LEPR)
8863	PER3	Homo sapiens	period circadian clock 3(PER3)
3298	HSF2	Homo sapiens	heat shock transcription factor 2(HSF2)
7130	TNFAIP6	Homo sapiens	TNF alpha induced protein 6(TNFAIP6)
563	AZGP1	Homo sapiens	alpha-2-glycoprotein 1, zinc-binding(AZGP1)
3569	IL6	Homo sapiens	interleukin 6(IL6)
1301	COL11A1	Homo sapiens	collagen type XI alpha 1 chain(COL11A1)
55655	NLRP2	Homo sapiens	NLR family pyrin domain containing 2(NLRP2)
3202	HOXA5	Homo sapiens	homeobox A5(HOXA5)
3201	HOXA4	Homo sapiens	homeobox A4(HOXA4)
84870	rspo3	Homo sapiens	R-spondin 3(RSPO3)
4015	LOX	Homo sapiens	lysyl oxidase(LOX)
10462	CLEC10A	Homo sapiens	C-type lectin domain family 10 member A(CLEC10A)
6039	RNASE6	Homo sapiens	ribonuclease A family member k6(RNASE6)
4016	LOXL1	Homo sapiens	lysyl oxidase like 1(LOXL1)
1949	EFNB3	Homo sapiens	ephrin B3(EFNB3)

969	CD69	Homo sapiens	CD69 molecule(CD69)
1306	COL15A1	Homo sapiens	collagen type XV alpha 1 chain(COL15A1)
7021	TFAP2B	Homo sapiens	transcription factor AP-2 beta(TFAP2B)
8111	GPR68	Homo sapiens	G protein-coupled receptor 68(GPR68)
7025	NR2F1	Homo sapiens	nuclear receptor subfamily 2 group F member 1(NR2F1)
3340	NDST1	Homo sapiens	N-deacetylase and N-sulfotransferase 1(NDST1)
80223	RAB11FIP1	Homo sapiens	RAB11 family interacting protein 1(RAB11FIP1)
55662	HIF1AN	Homo sapiens	hypoxia inducible factor 1 alpha subunit inhibitor(HIF1AN)
1436	CSF1R	Homo sapiens	colony stimulating factor 1 receptor(CSF1R)
2524	FUT2	Homo sapiens	fucosyltransferase 2(FUT2)
972	CD74	Homo sapiens	CD74 molecule(CD74)
10319	LAMC3	Homo sapiens	laminin subunit gamma 3(LAMC3)
1278	COL1A2	Homo sapiens	collagen type I alpha 2 chain(COL1A2)
2123	EVI2A	Homo sapiens	ecotropic viral integration site 2A(EVI2A)
1397	CRIP2	Homo sapiens	cysteine rich protein 2(CRIP2)
57720	GPR107	Homo sapiens	G protein-coupled receptor 107(GPR107)
3212	HOXB2	Homo sapiens	homeobox B2(HOXB2)
51621	KLF13	Homo sapiens	Kruppel like factor 13(KLF13)
10673	TNFSF13B	Homo sapiens	tumor necrosis factor superfamily member 13b(TNFSF13B)
978	CDA	Homo sapiens	cytidine deaminase(CDA)
5999	RGS4	Homo sapiens	regulator of G-protein signaling 4(RGS4)
3976	LIF	Homo sapiens	leukemia inhibitory factor(LIF)
284110	GSDMA	Homo sapiens	gasdermin A(GSDMA)
345275	HSD17B13	Homo sapiens	hydroxysteroid 17-beta dehydrogenase 13(HSD17B13)
9332	CD163	Homo sapiens	CD163 molecule(CD163)
11095	ADAMTS8	Homo sapiens	ADAM metallopeptidase with thrombospondin type 1 motif 8(ADAMTS8)
164656	tmprss6	Homo sapiens	transmembrane protease, serine 6(TMPRSS6)
10163	WASF2	Homo sapiens	WAS protein family member 2(WASF2)
4321	MMP12	Homo sapiens	matrix metallopeptidase 12(MMP12)
3199	HOXA2	Homo sapiens	homeobox A2(HOXA2)
57452	GALNT16	Homo sapiens	polypeptide N-acetylgalactosaminyltransferase 16(GALNT16)
1291	COL6A1	Homo sapiens	collagen type VI alpha 1 chain(COL6A1)
57613	FAM234B	Homo sapiens	family with sequence similarity 234 member B(FAM234B)

80331	DNAJC5	Homo sapiens	DnaJ heat shock protein family (Hsp40) member C5(DNAJC5)
151516	ASPRV1	Homo sapiens	aspartic peptidase, retroviral-like 1(ASPRV1)
1289	COL5A1	Homo sapiens	collagen type V alpha 1 chain(COL5A1)
1047	CLGN	Homo sapiens	calmegin(CLGN)
4314	MMP3	Homo sapiens	matrix metallopeptidase 3(MMP3)
4318	MMP9	Homo sapiens	matrix metallopeptidase 9(MMP9)

Table S5. The skin tissue-specific expression protein network containing 907 proteins (.xlsx).

Tissue	NCBI-entrzeID	Z-score
Skin_specific_genes	729240	5.385165
Skin_specific_genes	100129271	5.38516
Skin_specific_genes	353131	5.385154
Skin_specific_genes	3849	5.385153
Skin_specific_genes	26239	5.385152
Skin_specific_genes	448835	5.385149
Skin_specific_genes	353134	5.385147
Skin_specific_genes	353132	5.385144
Skin_specific_genes	353140	5.385121
Skin_specific_genes	353141	5.385118
Skin_specific_genes	353137	5.385115
Skin_specific_genes	353133	5.385102
Skin_specific_genes	353139	5.385025
Skin_specific_genes	388698	5.38496
Skin_specific_genes	145264	5.384702
Skin_specific_genes	374454	5.384543
Skin_specific_genes	386681	5.38442
Skin_specific_genes	386678	5.384072
Skin_specific_genes	653240	5.384068
Skin_specific_genes	386682	5.383965
Skin_specific_genes	254910	5.383926
Skin_specific_genes	353333	5.383835
Skin_specific_genes	386680	5.383796
Skin_specific_genes	386676	5.383767
Skin_specific_genes	4014	5.383523
Skin_specific_genes	1041	5.383509

Skin_specific_genes	353323	5.383196
Skin_specific_genes	386679	5.383177
Skin_specific_genes	84639	5.382981
Skin_specific_genes	388555	5.382931
Skin_specific_genes	347516	5.382926
Skin_specific_genes	338322	5.382873
Skin_specific_genes	85289	5.382538
Skin_specific_genes	386683	5.382161
Skin_specific_genes	353332	5.382136
Skin_specific_genes	387836	5.381863
Skin_specific_genes	386685	5.380989
Skin_specific_genes	387266	5.380965
Skin_specific_genes	4759	5.380456
Skin_specific_genes	386677	5.380134
Skin_specific_genes	151516	5.380093
Skin_specific_genes	149708	5.379877
Skin_specific_genes	27178	5.379822
Skin_specific_genes	284486	5.379735
Skin_specific_genes	2312	5.379661
Skin_specific_genes	1823	5.379554
Skin_specific_genes	85290	5.379547
Skin_specific_genes	5673	5.379125
Skin_specific_genes	100505753	5.379109
Skin_specific_genes	386675	5.378776
Skin_specific_genes	284110	5.377694
Skin_specific_genes	100507608	5.377638
Skin_specific_genes	388818	5.377248
Skin_specific_genes	728255	5.376976
Skin_specific_genes	338785	5.376237
Skin_specific_genes	100505724	5.375779
Skin_specific_genes	85293	5.37575
Skin_specific_genes	8710	5.375601
Skin_specific_genes	344752	5.37509
Skin_specific_genes	83902	5.374875
Skin_specific_genes	199834	5.374111
Skin_specific_genes	7299	5.373936
Skin_specific_genes	100132386	5.372992
Skin_specific_genes	81870	5.372659

Skin_specific_genes	59344	5.372524
Skin_specific_genes	83900	5.372504
Skin_specific_genes	81850	5.372501
Skin_specific_genes	85280	5.372415
Skin_specific_genes	22798	5.372357
Skin_specific_genes	728224	5.371882
Skin_specific_genes	387267	5.371185
Skin_specific_genes	643803	5.371043
Skin_specific_genes	83755	5.37038
Skin_specific_genes	448834	5.370328
Skin_specific_genes	85291	5.370004
Skin_specific_genes	83899	5.369228
Skin_specific_genes	6706	5.368986
Skin_specific_genes	2315	5.368978
Skin_specific_genes	84616	5.368595
Skin_specific_genes	83901	5.368015
Skin_specific_genes	100132476	5.366701
Skin_specific_genes	81871	5.366243
Skin_specific_genes	730755	5.365555
Skin_specific_genes	81851	5.364806
Skin_specific_genes	85285	5.363657
Skin_specific_genes	81872	5.361914
Skin_specific_genes	23581	5.361614
Skin_specific_genes	3888	5.361152
Skin_specific_genes	83895	5.359807
Skin_specific_genes	80740	5.359746
Skin_specific_genes	83896	5.359105
Skin_specific_genes	158833	5.358558
Skin_specific_genes	1474	5.35561
Skin_specific_genes	79097	5.354962
Skin_specific_genes	728279	5.352789
Skin_specific_genes	3858	5.352707
Skin_specific_genes	83897	5.352428
Skin_specific_genes	3884	5.352137
Skin_specific_genes	10850	5.352128
Skin_specific_genes	85294	5.351434
Skin_specific_genes	25833	5.35102
Skin_specific_genes	353135	5.35034

Skin_specific_genes	125981	5.346889
Skin_specific_genes	58530	5.34655
Skin_specific_genes	127534	5.345593
Skin_specific_genes	147183	5.344447
Skin_specific_genes	3886	5.341442
Skin_specific_genes	8688	5.338102
Skin_specific_genes	439915	5.337282
Skin_specific_genes	1747	5.334777
Skin_specific_genes	254240	5.333741
Skin_specific_genes	9119	5.332399
Skin_specific_genes	59082	5.332211
Skin_specific_genes	319101	5.330017
Skin_specific_genes	93099	5.329006
Skin_specific_genes	123745	5.326474
Skin_specific_genes	765	5.325012
Skin_specific_genes	3229	5.324785
Skin_specific_genes	342574	5.323952
Skin_specific_genes	3891	5.323607
Skin_specific_genes	22943	5.319798
Skin_specific_genes	3887	5.313568
Skin_specific_genes	162605	5.308044
Skin_specific_genes	100431172	5.306086
Skin_specific_genes	3892	5.305789
Skin_specific_genes	51806	5.299313
Skin_specific_genes	3885	5.297719
Skin_specific_genes	112802	5.294732
Skin_specific_genes	337880	5.290362
Skin_specific_genes	10089	5.289211
Skin_specific_genes	3881	5.289194
Skin_specific_genes	163778	5.288473
Skin_specific_genes	2201	5.286916
Skin_specific_genes	5676	5.282876
Skin_specific_genes	1638	5.281496
Skin_specific_genes	646480	5.276618
Skin_specific_genes	1828	5.271966
Skin_specific_genes	50489	5.269941
Skin_specific_genes	126637	5.267608
Skin_specific_genes	390792	5.262973

Skin_specific_genes	221301	5.261148
Skin_specific_genes	340654	5.260727
Skin_specific_genes	84560	5.259705
Skin_specific_genes	170680	5.257055
Skin_specific_genes	116093	5.254349
Skin_specific_genes	27177	5.254308
Skin_specific_genes	121391	5.252658
Skin_specific_genes	3848	5.252263
Skin_specific_genes	574414	5.249314
Skin_specific_genes	3889	5.236629
Skin_specific_genes	653499	5.232241
Skin_specific_genes	7062	5.225897
Skin_specific_genes	100533177	5.225703
Skin_specific_genes	158835	5.217831
Skin_specific_genes	768239	5.21537
Skin_specific_genes	29094	5.210402
Skin_specific_genes	10816	5.209325
Skin_specific_genes	643414	5.194081
Skin_specific_genes	242	5.192114
Skin_specific_genes	145447	5.192065
Skin_specific_genes	92211	5.189915
Skin_specific_genes	55076	5.188226
Skin_specific_genes	64711	5.184506
Skin_specific_genes	337879	5.178734
Skin_specific_genes	25818	5.171835
Skin_specific_genes	284353	5.171276
Skin_specific_genes	117159	5.169243
Skin_specific_genes	126767	5.168639
Skin_specific_genes	8687	5.164837
Skin_specific_genes	5069	5.159637
Skin_specific_genes	642	5.157198
Skin_specific_genes	3861	5.151901
Skin_specific_genes	3883	5.151593
Skin_specific_genes	8560	5.149308
Skin_specific_genes	26154	5.147219
Skin_specific_genes	3882	5.139939
Skin_specific_genes	337972	5.136695
Skin_specific_genes	285848	5.126277

Skin_specific_genes	83401	5.122788
Skin_specific_genes	5672	5.120834
Skin_specific_genes	909	5.11658
Skin_specific_genes	147920	5.106316
Skin_specific_genes	353288	5.091911
Skin_specific_genes	144501	5.089431
Skin_specific_genes	1046	5.085485
Skin_specific_genes	386674	5.085452
Skin_specific_genes	126917	5.083817
Skin_specific_genes	343641	5.082984
Skin_specific_genes	56998	5.082077
Skin_specific_genes	79983	5.08194
Skin_specific_genes	5669	5.081301
Skin_specific_genes	119548	5.078213
Skin_specific_genes	7045	5.045595
Skin_specific_genes	644054	5.045055
Skin_specific_genes	26525	5.032781
Skin_specific_genes	5806	5.023553
Skin_specific_genes	147700	5.018426
Skin_specific_genes	9333	5.015926
Skin_specific_genes	8139	5.012053
Skin_specific_genes	2250	5.010577
Skin_specific_genes	5650	5.004027
Skin_specific_genes	284348	4.992381
Skin_specific_genes	3963	4.991501
Skin_specific_genes	2707	4.986588
Skin_specific_genes	3120	4.975187
Skin_specific_genes	1308	4.957913
Skin_specific_genes	7306	4.955622
Skin_specific_genes	29113	4.950728
Skin_specific_genes	327657	4.942233
Skin_specific_genes	1775	4.939905
Skin_specific_genes	390093	4.934675
Skin_specific_genes	23086	4.925471
Skin_specific_genes	1749	4.905687
Skin_specific_genes	56300	4.898485
Skin_specific_genes	8091	4.890896
Skin_specific_genes	8456	4.87563

Skin_specific_genes	388533	4.836554
Skin_specific_genes	2335	4.824481
Skin_specific_genes	6274	4.798103
Skin_specific_genes	55507	4.793016
Skin_specific_genes	7286	4.790054
Skin_specific_genes	23087	4.772281
Skin_specific_genes	79850	4.766727
Skin_specific_genes	391123	4.76583
Skin_specific_genes	3857	4.74925
Skin_specific_genes	374897	4.748134
Skin_specific_genes	337882	4.740041
Skin_specific_genes	337959	4.738457
Skin_specific_genes	5670	4.733454
Skin_specific_genes	54346	4.730499
Skin_specific_genes	6785	4.71714
Skin_specific_genes	1944	4.716644
Skin_specific_genes	3228	4.705267
Skin_specific_genes	11247	4.702307
Skin_specific_genes	27006	4.692312
Skin_specific_genes	255189	4.686344
Skin_specific_genes	5317	4.677626
Skin_specific_genes	1825	4.669751
Skin_specific_genes	4311	4.645963
Skin_specific_genes	11219	4.643009
Skin_specific_genes	131578	4.626626
Skin_specific_genes	55806	4.623094
Skin_specific_genes	2019	4.610737
Skin_specific_genes	337971	4.606697
Skin_specific_genes	7499	4.603045
Skin_specific_genes	7473	4.588378
Skin_specific_genes	145226	4.573504
Skin_specific_genes	340745	4.566935
Skin_specific_genes	3227	4.525361
Skin_specific_genes	121214	4.516122
Skin_specific_genes	57864	4.501684
Skin_specific_genes	402415	4.481992
Skin_specific_genes	83481	4.478589
Skin_specific_genes	84525	4.47475

Skin_specific_genes	348938	4.465501
Skin_specific_genes	644815	4.463016
Skin_specific_genes	84000	4.445278
Skin_specific_genes	5268	4.44223
Skin_specific_genes	115908	4.420661
Skin_specific_genes	128876	4.416024
Skin_specific_genes	1832	4.414518
Skin_specific_genes	148741	4.412441
Skin_specific_genes	337974	4.411246
Skin_specific_genes	30814	4.399674
Skin_specific_genes	147409	4.380598
Skin_specific_genes	5542	4.374621
Skin_specific_genes	260434	4.372315
Skin_specific_genes	337966	4.352123
Skin_specific_genes	140258	4.336333
Skin_specific_genes	55584	4.324904
Skin_specific_genes	5271	4.323187
Skin_specific_genes	2117	4.307187
Skin_specific_genes	6095	4.293361
Skin_specific_genes	29986	4.283232
Skin_specific_genes	135228	4.282153
Skin_specific_genes	4015	4.281488
Skin_specific_genes	3416	4.271915
Skin_specific_genes	79412	4.267588
Skin_specific_genes	386684	4.253499
Skin_specific_genes	389336	4.250502
Skin_specific_genes	64065	4.240789
Skin_specific_genes	337973	4.240305
Skin_specific_genes	92196	4.220111
Skin_specific_genes	1301	4.216293
Skin_specific_genes	1748	4.216104
Skin_specific_genes	79755	4.210189
Skin_specific_genes	254773	4.208226
Skin_specific_genes	57152	4.205203
Skin_specific_genes	128488	4.202673
Skin_specific_genes	9076	4.192387
Skin_specific_genes	353144	4.190598
Skin_specific_genes	126410	4.180918

Skin_specific_genes	4861	4.173582
Skin_specific_genes	53833	4.163579
Skin_specific_genes	80834	4.156467
Skin_specific_genes	84951	4.123195
Skin_specific_genes	337963	4.118335
Skin_specific_genes	64919	4.11318
Skin_specific_genes	2261	4.10476
Skin_specific_genes	113146	4.098857
Skin_specific_genes	2709	4.097515
Skin_specific_genes	256227	4.093451
Skin_specific_genes	10793	4.088782
Skin_specific_genes	84290	4.083425
Skin_specific_genes	7020	4.07808
Skin_specific_genes	9314	4.074784
Skin_specific_genes	222584	4.067642
Skin_specific_genes	51200	4.066572
Skin_specific_genes	2625	4.063654
Skin_specific_genes	28976	4.059651
Skin_specific_genes	9572	4.036544
Skin_specific_genes	80352	4.031122
Skin_specific_genes	337970	4.026447
Skin_specific_genes	167127	4.01732
Skin_specific_genes	4038	4.017262
Skin_specific_genes	200424	3.988465
Skin_specific_genes	23299	3.981343
Skin_specific_genes	9610	3.978836
Skin_specific_genes	84975	3.978362
Skin_specific_genes	149428	3.977375
Skin_specific_genes	728318	3.968937
Skin_specific_genes	2617	3.967881
Skin_specific_genes	3037	3.962316
Skin_specific_genes	337976	3.961534
Skin_specific_genes	79852	3.960207
Skin_specific_genes	26585	3.959305
Skin_specific_genes	10848	3.940642
Skin_specific_genes	389119	3.92171
Skin_specific_genes	222	3.916294
Skin_specific_genes	4193	3.911828

Skin_specific_genes	387273	3.901595
Skin_specific_genes	100131897	3.873379
Skin_specific_genes	3728	3.866397
Skin_specific_genes	150094	3.863293
Skin_specific_genes	80329	3.861859
Skin_specific_genes	479	3.861029
Skin_specific_genes	8808	3.855402
Skin_specific_genes	51499	3.84607
Skin_specific_genes	23650	3.841986
Skin_specific_genes	51195	3.839787
Skin_specific_genes	2051	3.828986
Skin_specific_genes	162514	3.824963
Skin_specific_genes	55294	3.817559
Skin_specific_genes	11072	3.816432
Skin_specific_genes	55504	3.793312
Skin_specific_genes	51554	3.782804
Skin_specific_genes	2825	3.782307
Skin_specific_genes	123099	3.781591
Skin_specific_genes	55304	3.769698
Skin_specific_genes	204219	3.759909
Skin_specific_genes	79669	3.736215
Skin_specific_genes	93517	3.735788
Skin_specific_genes	80320	3.731799
Skin_specific_genes	152519	3.729055
Skin_specific_genes	43847	3.728226
Skin_specific_genes	50487	3.725131
Skin_specific_genes	11202	3.724464
Skin_specific_genes	595	3.713799
Skin_specific_genes	728113	3.702976
Skin_specific_genes	26496	3.698236
Skin_specific_genes	83541	3.665753
Skin_specific_genes	2205	3.661351
Skin_specific_genes	25946	3.659724
Skin_specific_genes	50604	3.658007
Skin_specific_genes	4017	3.656186
Skin_specific_genes	26219	3.651288
Skin_specific_genes	57662	3.642559
Skin_specific_genes	54602	3.634982

Skin_specific_genes	8626	3.632004
Skin_specific_genes	6374	3.630833
Skin_specific_genes	11187	3.620333
Skin_specific_genes	9547	3.620089
Skin_specific_genes	378884	3.594564
Skin_specific_genes	645402	3.59136
Skin_specific_genes	284207	3.585409
Skin_specific_genes	11166	3.58474
Skin_specific_genes	51384	3.57537
Skin_specific_genes	4312	3.562283
Skin_specific_genes	10248	3.560823
Skin_specific_genes	9697	3.556588
Skin_specific_genes	3664	3.547361
Skin_specific_genes	130576	3.539113
Skin_specific_genes	416	3.533999
Skin_specific_genes	441911	3.533079
Skin_specific_genes	57799	3.531809
Skin_specific_genes	3890	3.50869
Skin_specific_genes	53832	3.50616
Skin_specific_genes	158131	3.501386
Skin_specific_genes	245973	3.498806
Skin_specific_genes	8434	3.492212
Skin_specific_genes	84552	3.488419
Skin_specific_genes	8061	3.486814
Skin_specific_genes	64834	3.47832
Skin_specific_genes	92	3.471613
Skin_specific_genes	114771	3.456737
Skin_specific_genes	117194	3.455379
Skin_specific_genes	64768	3.446922
Skin_specific_genes	100191040	3.44592
Skin_specific_genes	11245	3.436059
Skin_specific_genes	23219	3.433839
Skin_specific_genes	2069	3.431436
Skin_specific_genes	345274	3.430948
Skin_specific_genes	11015	3.427129
Skin_specific_genes	150379	3.426462
Skin_specific_genes	10928	3.425295
Skin_specific_genes	6199	3.410545

Skin_specific_genes	92126	3.410334
Skin_specific_genes	337975	3.40626
Skin_specific_genes	79690	3.402943
Skin_specific_genes	79092	3.402905
Skin_specific_genes	51228	3.396588
Skin_specific_genes	126433	3.394527
Skin_specific_genes	59345	3.386242
Skin_specific_genes	389541	3.362626
Skin_specific_genes	1984	3.356157
Skin_specific_genes	3872	3.353508
Skin_specific_genes	1956	3.332515
Skin_specific_genes	120066	3.325235
Skin_specific_genes	121457	3.324667
Skin_specific_genes	81706	3.318506
Skin_specific_genes	2829	3.303383
Skin_specific_genes	144110	3.298834
Skin_specific_genes	2200	3.297183
Skin_specific_genes	10644	3.295371
Skin_specific_genes	100133093	3.294968
Skin_specific_genes	1278	3.292581
Skin_specific_genes	84962	3.284782
Skin_specific_genes	643418	3.284709
Skin_specific_genes	133	3.282158
Skin_specific_genes	1960	3.280655
Skin_specific_genes	54733	3.277169
Skin_specific_genes	91694	3.276331
Skin_specific_genes	23596	3.268567
Skin_specific_genes	91	3.263569
Skin_specific_genes	1303	3.256857
Skin_specific_genes	2058	3.244193
Skin_specific_genes	580	3.241957
Skin_specific_genes	3159	3.237314
Skin_specific_genes	112724	3.232117
Skin_specific_genes	6059	3.230853
Skin_specific_genes	133060	3.227849
Skin_specific_genes	29904	3.220427
Skin_specific_genes	55038	3.216801
Skin_specific_genes	80157	3.208788

Skin_specific_genes	3852	3.206552
Skin_specific_genes	54407	3.201824
Skin_specific_genes	4308	3.20055
Skin_specific_genes	7291	3.199163
Skin_specific_genes	148304	3.194234
Skin_specific_genes	118430	3.191833
Skin_specific_genes	84549	3.179723
Skin_specific_genes	132014	3.173541
Skin_specific_genes	51513	3.173357
Skin_specific_genes	84069	3.169442
Skin_specific_genes	23471	3.166655
Skin_specific_genes	2049	3.166637
Skin_specific_genes	9692	3.160848
Skin_specific_genes	8689	3.158895
Skin_specific_genes	3376	3.158855
Skin_specific_genes	122786	3.152035
Skin_specific_genes	84876	3.142693
Skin_specific_genes	4610	3.141377
Skin_specific_genes	23682	3.140065
Skin_specific_genes	160418	3.129357
Skin_specific_genes	7022	3.126915
Skin_specific_genes	1300	3.125648
Skin_specific_genes	644943	3.125288
Skin_specific_genes	338773	3.11317
Skin_specific_genes	79670	3.109135
Skin_specific_genes	8784	3.10348
Skin_specific_genes	10647	3.100428
Skin_specific_genes	6536	3.098657
Skin_specific_genes	402635	3.097217
Skin_specific_genes	1945	3.094581
Skin_specific_genes	728458	3.092356
Skin_specific_genes	100288323	3.085231
Skin_specific_genes	10643	3.082025
Skin_specific_genes	57115	3.073664
Skin_specific_genes	9949	3.059176
Skin_specific_genes	80765	3.055284
Skin_specific_genes	2191	3.04745
Skin_specific_genes	112399	3.047177

Skin_specific_genes	6591	3.047024
Skin_specific_genes	9651	3.04316
Skin_specific_genes	6704	3.023992
Skin_specific_genes	538	3.022752
Skin_specific_genes	5916	3.01886
Skin_specific_genes	6541	3.018027
Skin_specific_genes	8840	3.016836
Skin_specific_genes	2125	3.009433
Skin_specific_genes	344905	3.000027
Skin_specific_genes	80195	2.997373
Skin_specific_genes	1241	2.992061
Skin_specific_genes	1212	2.989373
Skin_specific_genes	57830	2.987971
Skin_specific_genes	11260	2.986775
Skin_specific_genes	80194	2.98277
Skin_specific_genes	342897	2.972949
Skin_specific_genes	195814	2.970847
Skin_specific_genes	5453	2.964694
Skin_specific_genes	89777	2.96384
Skin_specific_genes	10457	2.962667
Skin_specific_genes	27076	2.961311
Skin_specific_genes	5270	2.96048
Skin_specific_genes	401541	2.955209
Skin_specific_genes	391723	2.948838
Skin_specific_genes	11217	2.947318
Skin_specific_genes	1588	2.946516
Skin_specific_genes	302	2.943929
Skin_specific_genes	54897	2.94081
Skin_specific_genes	7477	2.930912
Skin_specific_genes	688	2.92803
Skin_specific_genes	160428	2.92614
Skin_specific_genes	2810	2.925137
Skin_specific_genes	55299	2.91844
Skin_specific_genes	201799	2.915055
Skin_specific_genes	10973	2.911317
Skin_specific_genes	5054	2.908639
Skin_specific_genes	117581	2.906857
Skin_specific_genes	339665	2.906226

Skin_specific_genes	56169	2.90455
Skin_specific_genes	389136	2.903589
Skin_specific_genes	6240	2.89703
Skin_specific_genes	7474	2.896018
Skin_specific_genes	5036	2.895851
Skin_specific_genes	84135	2.894759
Skin_specific_genes	171177	2.88969
Skin_specific_genes	3898	2.886643
Skin_specific_genes	960	2.88536
Skin_specific_genes	3224	2.880839
Skin_specific_genes	136	2.879174
Skin_specific_genes	92667	2.878977
Skin_specific_genes	8416	2.876766
Skin_specific_genes	54989	2.871284
Skin_specific_genes	25984	2.870165
Skin_specific_genes	341277	2.868262
Skin_specific_genes	196264	2.867128
Skin_specific_genes	7371	2.844173
Skin_specific_genes	913	2.838242
Skin_specific_genes	79191	2.830667
Skin_specific_genes	7057	2.830304
Skin_specific_genes	2247	2.822015
Skin_specific_genes	345651	2.816891
Skin_specific_genes	7980	2.813902
Skin_specific_genes	53836	2.807651
Skin_specific_genes	10226	2.804375
Skin_specific_genes	120065	2.799443
Skin_specific_genes	84262	2.794548
Skin_specific_genes	80346	2.789198
Skin_specific_genes	222894	2.788715
Skin_specific_genes	81555	2.78379
Skin_specific_genes	26472	2.78308
Skin_specific_genes	493869	2.782643
Skin_specific_genes	3265	2.780943
Skin_specific_genes	6820	2.779453
Skin_specific_genes	813	2.777412
Skin_specific_genes	381	2.774108
Skin_specific_genes	50805	2.772848

Skin_specific_genes	79993	2.768086
Skin_specific_genes	5352	2.768035
Skin_specific_genes	81565	2.760816
Skin_specific_genes	51388	2.758695
Skin_specific_genes	80262	2.755363
Skin_specific_genes	9420	2.753697
Skin_specific_genes	8796	2.752141
Skin_specific_genes	8793	2.750621
Skin_specific_genes	267020	2.750159
Skin_specific_genes	10451	2.750144
Skin_specific_genes	9721	2.749803
Skin_specific_genes	8038	2.746711
Skin_specific_genes	440050	2.742661
Skin_specific_genes	3156	2.741858
Skin_specific_genes	337977	2.738943
Skin_specific_genes	115572	2.738788
Skin_specific_genes	63970	2.730345
Skin_specific_genes	9554	2.730041
Skin_specific_genes	10970	2.727938
Skin_specific_genes	5163	2.727363
Skin_specific_genes	53335	2.726875
Skin_specific_genes	80045	2.725541
Skin_specific_genes	55223	2.722588
Skin_specific_genes	1293	2.717655
Skin_specific_genes	55561	2.712001
Skin_specific_genes	7984	2.710584
Skin_specific_genes	8581	2.707115
Skin_specific_genes	55752	2.706741
Skin_specific_genes	84283	2.702095
Skin_specific_genes	3606	2.698271
Skin_specific_genes	4004	2.688661
Skin_specific_genes	26225	2.687968
Skin_specific_genes	23160	2.676724
Skin_specific_genes	729830	2.672729
Skin_specific_genes	79190	2.671861
Skin_specific_genes	2773	2.670682
Skin_specific_genes	54361	2.670666
Skin_specific_genes	63933	2.663701

Skin_specific_genes	6050	2.659518
Skin_specific_genes	643853	2.65913
Skin_specific_genes	7465	2.658441
Skin_specific_genes	5917	2.656164
Skin_specific_genes	1277	2.649187
Skin_specific_genes	8614	2.648057
Skin_specific_genes	5118	2.646786
Skin_specific_genes	6731	2.644173
Skin_specific_genes	147372	2.636766
Skin_specific_genes	6176	2.626454
Skin_specific_genes	123169	2.621632
Skin_specific_genes	4285	2.621054
Skin_specific_genes	121665	2.62039
Skin_specific_genes	90956	2.607658
Skin_specific_genes	8411	2.606904
Skin_specific_genes	1678	2.60464
Skin_specific_genes	54510	2.603423
Skin_specific_genes	2967	2.601508
Skin_specific_genes	65985	2.600096
Skin_specific_genes	6382	2.596261
Skin_specific_genes	147495	2.595114
Skin_specific_genes	1290	2.594927
Skin_specific_genes	23276	2.593953
Skin_specific_genes	5279	2.592941
Skin_specific_genes	3692	2.592247
Skin_specific_genes	24145	2.591297
Skin_specific_genes	10009	2.590888
Skin_specific_genes	283489	2.584717
Skin_specific_genes	1294	2.584399
Skin_specific_genes	91298	2.576858
Skin_specific_genes	3068	2.574204
Skin_specific_genes	114990	2.571497
Skin_specific_genes	5887	2.567646
Skin_specific_genes	125988	2.566919
Skin_specific_genes	26135	2.565457
Skin_specific_genes	9358	2.561177
Skin_specific_genes	55181	2.55999
Skin_specific_genes	1849	2.55906

Skin_specific_genes	151887	2.558681
Skin_specific_genes	390	2.558488
Skin_specific_genes	5017	2.556629
Skin_specific_genes	27042	2.553206
Skin_specific_genes	90060	2.551132
Skin_specific_genes	653145	2.551109
Skin_specific_genes	100137047	2.548886
Skin_specific_genes	6455	2.541814
Skin_specific_genes	283951	2.540376
Skin_specific_genes	29899	2.530895
Skin_specific_genes	79828	2.524912
Skin_specific_genes	10606	2.524577
Skin_specific_genes	59269	2.522206
Skin_specific_genes	6665	2.515686
Skin_specific_genes	115825	2.51347
Skin_specific_genes	85465	2.512573
Skin_specific_genes	5784	2.502747
Skin_specific_genes	1215	2.502283
Skin_specific_genes	57168	2.501688
Skin_specific_genes	10424	2.494734
Skin_specific_genes	4000	2.491615
Skin_specific_genes	339230	2.491458
Skin_specific_genes	653464	2.487506
Skin_specific_genes	57449	2.487346
Skin_specific_genes	10492	2.475514
Skin_specific_genes	9329	2.472956
Skin_specific_genes	54478	2.469823
Skin_specific_genes	400506	2.467996
Skin_specific_genes	64066	2.467082
Skin_specific_genes	1643	2.466698
Skin_specific_genes	158219	2.462612
Skin_specific_genes	11174	2.46086
Skin_specific_genes	11012	2.457726
Skin_specific_genes	128272	2.451857
Skin_specific_genes	163259	2.447438
Skin_specific_genes	51334	2.438834
Skin_specific_genes	4893	2.437407
Skin_specific_genes	3770	2.435718

Skin_specific_genes	54845	2.434446
Skin_specific_genes	647024	2.43299
Skin_specific_genes	6701	2.429455
Skin_specific_genes	430	2.426088
Skin_specific_genes	84659	2.423936
Skin_specific_genes	57216	2.412955
Skin_specific_genes	134957	2.410174
Skin_specific_genes	25921	2.4098
Skin_specific_genes	29968	2.408246
Skin_specific_genes	2589	2.395038
Skin_specific_genes	63943	2.392431
Skin_specific_genes	51154	2.392384
Skin_specific_genes	11221	2.389199
Skin_specific_genes	4313	2.387833
Skin_specific_genes	55207	2.387326
Skin_specific_genes	4853	2.385041
Skin_specific_genes	337867	2.378855
Skin_specific_genes	4609	2.377861
Skin_specific_genes	80131	2.37612
Skin_specific_genes	6222	2.375615
Skin_specific_genes	10758	2.370696
Skin_specific_genes	51441	2.367413
Skin_specific_genes	165679	2.36687
Skin_specific_genes	131566	2.363929
Skin_specific_genes	54555	2.36258
Skin_specific_genes	5591	2.35929
Skin_specific_genes	54433	2.358913
Skin_specific_genes	1175	2.357589
Skin_specific_genes	5395	2.357521
Skin_specific_genes	26872	2.356326
Skin_specific_genes	55659	2.354415
Skin_specific_genes	9841	2.354001
Skin_specific_genes	10265	2.353711
Skin_specific_genes	64864	2.351903
Skin_specific_genes	5469	2.348081
Skin_specific_genes	51493	2.34796
Skin_specific_genes	6715	2.34315
Skin_specific_genes	55198	2.341812

Skin_specific_genes	6897	2.337898
Skin_specific_genes	23016	2.337786
Skin_specific_genes	642475	2.333704
Skin_specific_genes	382	2.333082
Skin_specific_genes	200916	2.329612
Skin_specific_genes	55920	2.323922
Skin_specific_genes	57333	2.322783
Skin_specific_genes	51450	2.321797
Skin_specific_genes	10197	2.319564
Skin_specific_genes	27315	2.316785
Skin_specific_genes	23077	2.315621
Skin_specific_genes	9183	2.315123
Skin_specific_genes	64393	2.309728
Skin_specific_genes	731220	2.308439
Skin_specific_genes	83640	2.307877
Skin_specific_genes	1021	2.305567
Skin_specific_genes	23624	2.303736
Skin_specific_genes	23233	2.301757
Skin_specific_genes	51114	2.299356
Skin_specific_genes	647135	2.297513
Skin_specific_genes	6301	2.297372
Skin_specific_genes	51043	2.296425
Skin_specific_genes	9536	2.296025
Skin_specific_genes	4931	2.292726
Skin_specific_genes	90850	2.292144
Skin_specific_genes	23431	2.287526
Skin_specific_genes	64121	2.283943
Skin_specific_genes	5361	2.281782
Skin_specific_genes	24147	2.2812
Skin_specific_genes	6175	2.280361
Skin_specific_genes	286077	2.276832
Skin_specific_genes	440021	2.273518
Skin_specific_genes	2760	2.27331
Skin_specific_genes	8785	2.271551
Skin_specific_genes	353	2.269137
Skin_specific_genes	89958	2.267676
Skin_specific_genes	145748	2.267661
Skin_specific_genes	23002	2.265782

Skin_specific_genes	643904	2.261138
Skin_specific_genes	57146	2.260916
Skin_specific_genes	23065	2.26023
Skin_specific_genes	57111	2.256296
Skin_specific_genes	445328	2.254713
Skin_specific_genes	29841	2.25469
Skin_specific_genes	388567	2.252281
Skin_specific_genes	51534	2.249552
Skin_specific_genes	833	2.24898
Skin_specific_genes	8535	2.247766
Skin_specific_genes	8568	2.246721
Skin_specific_genes	8754	2.242019
Skin_specific_genes	8428	2.238696
Skin_specific_genes	51808	2.237332
Skin_specific_genes	54869	2.235807
Skin_specific_genes	5753	2.233851
Skin_specific_genes	2963	2.233591
Skin_specific_genes	55651	2.231766
Skin_specific_genes	51151	2.230397
Skin_specific_genes	343578	2.228585
Skin_specific_genes	908	2.228078
Skin_specific_genes	90019	2.226747
Skin_specific_genes	6223	2.226734
Skin_specific_genes	2150	2.226343
Skin_specific_genes	23205	2.224401
Skin_specific_genes	81786	2.220927
Skin_specific_genes	5478	2.218051
Skin_specific_genes	2178	2.217853
Skin_specific_genes	3939	2.216928
Skin_specific_genes	83743	2.213256
Skin_specific_genes	10458	2.212467
Skin_specific_genes	5130	2.207367
Skin_specific_genes	387521	2.20564
Skin_specific_genes	284098	2.204862
Skin_specific_genes	389558	2.203924
Skin_specific_genes	10631	2.201613
Skin_specific_genes	63928	2.199753
Skin_specific_genes	9509	2.199172

Skin_specific_genes	25832	2.19457
Skin_specific_genes	339175	2.194417
Skin_specific_genes	8894	2.192979
Skin_specific_genes	3918	2.191491
Skin_specific_genes	56603	2.18625
Skin_specific_genes	8323	2.183994
Skin_specific_genes	79797	2.183231
Skin_specific_genes	6712	2.180074
Skin_specific_genes	3226	2.1774
Skin_specific_genes	55536	2.177351
Skin_specific_genes	27113	2.176951
Skin_specific_genes	1946	2.176296
Skin_specific_genes	8602	2.175508
Skin_specific_genes	152002	2.174786
Skin_specific_genes	4323	2.174531
Skin_specific_genes	94137	2.174416
Skin_specific_genes	79939	2.17356
Skin_specific_genes	2107	2.169423
Skin_specific_genes	9188	2.169314
Skin_specific_genes	871	2.165975
Skin_specific_genes	55734	2.16401
Skin_specific_genes	6603	2.160819
Skin_specific_genes	55703	2.156295
Skin_specific_genes	11211	2.151677
Skin_specific_genes	26149	2.149044
Skin_specific_genes	10370	2.146222
Skin_specific_genes	204962	2.143686
Skin_specific_genes	1746	2.141702
Skin_specific_genes	5783	2.141438
Skin_specific_genes	124975	2.137918
Skin_specific_genes	29940	2.137799
Skin_specific_genes	54841	2.131462
Skin_specific_genes	653583	2.131193
Skin_specific_genes	60412	2.130656
Skin_specific_genes	9792	2.122001
Skin_specific_genes	84260	2.117746
Skin_specific_genes	3091	2.117413
Skin_specific_genes	57724	2.114893

Skin_specific_genes	23481	2.11405
Skin_specific_genes	5954	2.113061
Skin_specific_genes	57761	2.111755
Skin_specific_genes	57636	2.111615
Skin_specific_genes	4250	2.110278
Skin_specific_genes	79803	2.109908
Skin_specific_genes	285855	2.109892
Skin_specific_genes	154	2.10673
Skin_specific_genes	7508	2.105082
Skin_specific_genes	85450	2.103459
Skin_specific_genes	60370	2.103325
Skin_specific_genes	27	2.100597
Skin_specific_genes	11328	2.096976
Skin_specific_genes	125113	2.096639
Skin_specific_genes	22911	2.096383
Skin_specific_genes	9448	2.09552
Skin_specific_genes	5429	2.090374
Skin_specific_genes	9993	2.08478
Skin_specific_genes	6868	2.083404
Skin_specific_genes	84790	2.083202
Skin_specific_genes	81929	2.082973
Skin_specific_genes	1525	2.07773
Skin_specific_genes	26227	2.07736
Skin_specific_genes	6520	2.077273
Skin_specific_genes	57822	2.077003
Skin_specific_genes	51274	2.072012
Skin_specific_genes	1952	2.071509
Skin_specific_genes	705	2.069997
Skin_specific_genes	1026	2.065852
Skin_specific_genes	259266	2.06264
Skin_specific_genes	54918	2.061992
Skin_specific_genes	56915	2.061567
Skin_specific_genes	85456	2.06084
Skin_specific_genes	8661	2.058876
Skin_specific_genes	51491	2.057318
Skin_specific_genes	153572	2.056411
Skin_specific_genes	1174	2.05548
Skin_specific_genes	79071	2.055109

Skin_specific_genes	55798	2.053829
Skin_specific_genes	55356	2.051661
Skin_specific_genes	653247	2.05032
Skin_specific_genes	55247	2.048351
Skin_specific_genes	6624	2.046434
Skin_specific_genes	92421	2.045471
Skin_specific_genes	144108	2.044057
Skin_specific_genes	1736	2.043109
Skin_specific_genes	23172	2.043043
Skin_specific_genes	64866	2.04284
Skin_specific_genes	79183	2.04231
Skin_specific_genes	57609	2.041751
Skin_specific_genes	342909	2.040314
Skin_specific_genes	140459	2.03971
Skin_specific_genes	114882	2.037266
Skin_specific_genes	65062	2.036426
Skin_specific_genes	4691	2.034748
Skin_specific_genes	100462983	2.034619
Skin_specific_genes	6736	2.034198
Skin_specific_genes	161424	2.033239
Skin_specific_genes	6385	2.032092
Skin_specific_genes	23243	2.031894
Skin_specific_genes	1513	2.031533
Skin_specific_genes	8751	2.030347
Skin_specific_genes	7335	2.029583
Skin_specific_genes	55771	2.029521
Skin_specific_genes	54933	2.0285
Skin_specific_genes	10940	2.02837
Skin_specific_genes	55083	2.028306
Skin_specific_genes	121642	2.027807
Skin_specific_genes	8879	2.026567
Skin_specific_genes	3916	2.022534
Skin_specific_genes	143244	2.02045
Skin_specific_genes	23268	2.019696
Skin_specific_genes	64746	2.019148
Skin_specific_genes	2697	2.015398
Skin_specific_genes	90678	2.014328
Skin_specific_genes	54626	2.013348

Skin_specific_genes	100134934	2.012861
Skin_specific_genes	84879	2.012222
Skin_specific_genes	10017	2.005455
Skin_specific_genes	7421	2.004886
Skin_specific_genes	54994	2.002747
Skin_specific_genes	6502	2.001644
Skin_specific_genes	2055	2.000219

Table S6. Real-time PCR Primers.

Gene	Primer sequence-forward	Primer sequence-reverse
GAPDH	CCAAGGAGTAAGACCCCTGG	AGGGGAGATTCAGTGTGGTG
MMP-1	TGGGAGGCAAGTTGAAAAGC	CATCTGGGCTGCTTCATCAC
MMP-3	CCTGCTTTGTCC TTTGATGC	TGAGTCAATCCCTGGAAAGTC
Procollagen type I	CTCGAGGTGGACACCCT	CAGCTGGATGGCCACATCGG
iNOS	ACCCAAGGTCTACGTTCAGG	CGCACATCTCCGCAAATGTA
TNF-α	AGGGGA AATGAGAGACGCAA	TTCCCCATCTCTTGCCACAT
IL-6	CTCCTTCTCCACAAGCGCC	GCCGAAGAGCCCTCAGGC

Table S7. The 22 ginsenosides with significant shortest distance (D_{min}<0.2) to the set of known anti-photoaging ginsenosides.

Pubchem CID	Name	Smiles	Shortest distance
181573	20(R)-Ginsenodie Rg3	O1C(CO)C(O)C(O)C(OC2OC(CO)C(O)C(O)C2O)C1OC1CCC2(C(CCC3C2CC(O)C2(C)C3(CCC2C(O)(CCC=C(C)C)C)C)C1(C)C)C	0.002370871
432447	NSC308876	O1C(CO)C(O)C(O)C(OC2OC(CO)C(O)C(O)C2O)C1OC1CCC2(C(CCC3(C2CC(O)C2C3(CCC2C(OC2OC(CO)C(O)C(O)C2O)(CCC=C(C)C)C)C)C)C1(C)C)C	0.010510023
44584555	Gynosaponin S	O1C(CO)C(O)C(O)C(O)C1OC1CCC2(C(CCC3(C2CC(O)C2C3(CCC2C(OC2OC(COC3OC(CO)C(O)C(O)C3O)C(O)C(O)C2O)(CCC=C(C)C)C)C)C)C1(C)C)C	0.015294697

86289140	Gypenoside LXXV	O1C(COC2OC(CO)C(O)C(O)C2O)C(O)C(O)C(O)C1OC(CCC=C(C)C)(C)C1C2C(CC1)(C)C1(C(CC2O)C2(C(CC1)C(C)(C)C(O)CC2)C)C	0.0178612
9918692	Ginsenoside F2	O1C(CO)C(O)C(O)C(O)C1OC1CCC2(C(CCC3(C2CC(O)C2C3(CCC2C(OC2OC(CO)C(O)C(O)C2O)(CCC=C(C)C)C)C)C)C1(C)C)C	0.02634638
20839223	Ginsenoside Rh3	O1C(CO)C(O)C(O)C(O)C1OC1CCC2(C(CCC3(C2CC(O)C2C3(CCC2C(=CCC=C(C)C)C)C)C)C1(C)C)C	0.038878612
90472238	Ginsenoside Rk2	O1C(CO)C(O)C(O)C(O)C1OC1CCC2(C(CCC3(C2CC(O)C2C3(CCC2C(CCC=C(C)C)=C)C)C)C1(C)C)C	0.054841947
101887367	Pseudoginsenoside Rh2	O1C(CO)C(O)C(O)C(O)C1OC1CCC2(C(CCC3(C2CC(O)C2C3(CCC2C(=CCCC(O)(C)C)C)C)C)C1(C)C)C	0.070376992
146160065	Ginsenoside Mb	O1C(CO)C(O)C(O)C(O)C1OC1CCC2(C(CCC3(C2CC(O)C2C3(CCC2C(CCC=C(C)C)COC2OC(COC3OC(CO)C(O)C3O)C(O)C(O)C2O)C)C)C1(C)C)C	0.098505876
131751696	Ginsenoside I	O1C(CO)C(O)C(O)C(OC2OC(CO)C(O)C(O)C2O)C1OC1CCC2(C(CCC3(C2CC(O)C2C3(CCC2C(OC2OC(CO)C(O)C(O)C2O)(CCC(OO)C(C)=C)C)C)C)C1(C)C)C	0.101592358
122173224	(R) Ginsenoside Rh2	O1C(CO)C(O)C(O)C(O)C1OC1CCC2(C(CCC3C2CC(O)C2C(CCC23C)(C(O)(CCC=C(C)C)C)C)C1(C)C)C	0.115939704
14081290	(20R)-Ginsenoside Rh2	O1C(CO)C(O)C(O)C(O)C1OC1CCC2(C(CCC3(C2CC(O)C2C3(CCC2C(O)(CCC=C(C)C)C)C)C)C1(C)C)C	0.130329937
91895489	Ginsenoside Rg6	O1C(CO)C(O)C(O)C(OC2OC(C)C(O)C(O)C2O)C1OC1C2C(C)(C)C(O)CCC2(C2CC(O)C3C(CCC3C(CCC=C(C)C)=C)(C)C2(C1)C)C	0.150443362
11331683	Ginsenoside Mx	O1C(COC2OCC(O)C(O)C2O)C(O)C(O)C(O)C1OC(CCC=C(C)C)(C)C1C2C(CC1)(C)C1(C(CC2O)C2(C(CC1)C(C)(C)C(O)CC2)C)C	0.158719637
73758547	Notoginsenoside Fd	O1C(CO)C(O)C(O)C(O)C1OC1CCC2(C(CCC3(C2CC(O)C2C3(CCC2C(OC2OC(COC3OCC(O)C(O)C3O)C(O)C(O)C2O)(CCC=C(C)C)C)C)C)C1(C)C)C	0.166430172

9896928	Ginsenoside Mc	O1C(COC2OC(CO)C(O)C2O)C(O)C(O)C(O)C1OC(CCC=C(C)C)(C)C1C2C(CC1)(C)C1(C(CC2O)C2(C(CC1)C(C)(C)C(O)CC2)C)C	0.167077587
131751552	Ginsenoside III	O1C(CO)C(O)C(O)C(OC2OC(CO)C(O)C(O)C2O)C1OC1CCC2(C(CCC3(C2CC(O)C2C3(CCC2C(OC2OC(CO)C(O)C(O)C2O)(CCC(=O)C(C)=C)C)C)C)C1(C)C)C	0.178895731
85044013	Ginsenoside Rs1	O1C(CO)C(O)C(O)C(OC2OC(COC(=O)C)C(O)C(O)C2O)C1OC1CCC2(C(CCC3(C2CC(O)C2C3(CCC2C(OC2OC(COC3OCC(O)C(O)C3O)C(O)C(O)C2O)(CCC=C(C)C)C)C)C)C1(C)C)C	0.181315932
11550001	Ginsenoside-Rg5	O1C(CO)C(O)C(O)C(OC2OC(CO)C(O)C(O)C2O)C1OC1CCC2(C(CCC3(C2CC(O)C2C3(CCC2C(=CCC=C(C)C)C)C)C)C1(C)C)C	0.187214052
73157065	Ginsenoside Rs2	O1C(COC(=O)C)C(O)C(O)C(OC2OC(CO)C(O)C(O)C2O)C1OC1CCC2(C(CCC3(C2CC(O)C2C3(CCC2C(OC2OC(COC3OC(CO)C(O)C3O)C(O)C(O)C2O)(CCC=C(C)C)C)C)C)C1(C)C)C	0.188511535
85237385	Ginsenoside Rh5	O1C(CO)C(O)C(O)C(O)C1OC1C2C(C)(C)C(O)CCC2(C2CC(O)C3C(CCC3C(OC)(CCC=C(C)C)C)(C)C2(C1)C)C	0.193396881
10629247	Vina-ginsenoside R25	O1C(CO)C(O)C(O)C(O)C1OC1C2C(C)(C)C(O)CCC2(C2CC(O)C3C(CCC3C(OC3OC(CO)C(O)C(O)C3O)(CCC(=O)C(C)=C)C)(C)C2(C1)C)C	0.195799324
